# Comprehensive molecular, genomic and phenotypic analysis of a major clone of *Enterococcus faecalis* MLST ST40

**DOI:** 10.1186/s12864-015-1367-x

**Published:** 2015-03-12

**Authors:** Melanie Zischka, Carsten T Künne, Jochen Blom, Dominique Wobser, Türkân Sakιnç, Kerstin Schmidt-Hohagen, P Wojtek Dabrowski, Andreas Nitsche, Johannes Hübner, Torsten Hain, Trinad Chakraborty, Burkhard Linke, Alexander Goesmann, Sonja Voget, Rolf Daniel, Dietmar Schomburg, Rüdiger Hauck, Hafez M Hafez, Petra Tielen, Dieter Jahn, Margrete Solheim, Ewa Sadowy, Jesper Larsen, Lars B Jensen, Patricia Ruiz-Garbajosa, Dianelys Quiñones Pérez, Theresa Mikalsen, Jennifer Bender, Matthias Steglich, Ulrich Nübel, Wolfgang Witte, Guido Werner

**Affiliations:** Division of Nosocomial Pathogens and Antibiotic Resistances, Department of Infectious Diseases, Robert Koch Institute, Wernigerode Branch, Burgstr. 37, D-38855 Wernigerode, Germany; Present address: Institute for Pathology, Hannover Medical School (MHH), Hannover, Germany; Functional Genomics of Bacterial Pathogens, Institute for Medical Microbiology, Justus Liebig University Giessen and German Center for Infection Research (DZIF), Partner site Giessen-Marburg-Langen, Campus Giessen, Giessen, Germany; Max Planck Institute for Heart and Lung Research, Bad Nauheim, Germany; Center for Biotechnology (CeBiTec)/University of Bielefeld, Bielefeld, Germany; Institute for Bioinformatics and Systems Biology, Justus Liebig University Giessen, Giessen, Germany; Division of Infectious Diseases, Department of Medicine, University Hospital Freiburg, Freiburg, Germany; Institute for Biochemistry, Biotechnology and Bioinformatics, Technische Universität Braunschweig, Braunschweig, Germany; Robert Koch Institute, ZBS 1 Highly Pathogenic Viruses, Centre for Biological Threats and Special Pathogens, Berlin, Germany; Division of Pediatric Infectious Diseases, Hauner Children’s Hospital, Ludwig-Maximilians University Munich, Munich, Germany; Goettingen Genomics Laboratory, Georg August University, Goettingen, Germany; Department of Veterinary Medicine, Institute for Poultry Diseases, Free University Berlin, Berlin, Germany; Institute for Microbiology, Technische Universität Braunschweig, Braunschweig, Germany; Laboratory of Microbial Gene Technology and Food Microbiology, The Norwegian University of Life Sciences, Ås, Norway; National Medicines Institute, Warsaw, Poland; Statens Serum Institute, Copenhagen, Denmark; Division of Microbiology, National Food Institute, Danish Technical University, Copenhagen, Denmark; Department of Microbiology, Hospital Ramon y Cajal, Madrid, Spain; Instituto de Medicina Tropical Pedro Kourí, Servicio de Bacteriología-Micología, La Habana, Cuba; Department of Medical Biology, Faculty of Health Sciences, Research Group for Host Microbe Interactions, University of Tromsø, Tromsø, Norway; Leibniz-Institut DSMZ - Deutsche Sammlung von Mikrorganismen und Zellkulturen GmbH, Braunschweig, Germany

**Keywords:** *Enterococcus faecalis*, Whole genome, Esp, Pathogenicity island, Capsule

## Abstract

**Background:**

*Enterococcus faecalis* is a multifaceted microorganism known to act as a beneficial intestinal commensal bacterium. It is also a dreaded nosocomial pathogen causing life-threatening infections in hospitalised patients. Isolates of a distinct MLST type ST40 represent the most frequent strain type of this species, distributed worldwide and originating from various sources (animal, human, environmental) and different conditions (colonisation/infection). Since enterococci are known to be highly recombinogenic we determined to analyse the microevolution and niche adaptation of this highly distributed clonal type.

**Results:**

We compared a set of 42 ST40 isolates by assessing key molecular determinants, performing whole genome sequencing (WGS) and a number of phenotypic assays including resistance profiling, formation of biofilm and utilisation of carbon sources. We generated the first circular closed reference genome of *an E. faecalis* isolate D32 of animal origin and compared it with the genomes of other reference strains. D32 was used as a template for detailed WGS comparisons of high-quality draft genomes of 14 ST40 isolates. Genomic and phylogenetic analyses suggest a high level of similarity regarding the core genome, also demonstrated by similar carbon utilisation patterns. Distribution of known and putative virulence-associated genes did not differentiate between ST40 strains from a commensal and clinical background or an animal or human source. Further analyses of mobile genetic elements (MGE) revealed genomic diversity owed to: (1) a modularly structured pathogenicity island; (2) a site-specifically integrated and previously unknown genomic island of 138 kb in two strains putatively involved in exopolysaccharide synthesis; and (3) isolate-specific plasmid and phage patterns. Moreover, we used different cell-biological and animal experiments to compare the isolate D32 with a closely related ST40 endocarditis isolate whose draft genome sequence was also generated. D32 generally showed a greater capacity of adherence to human cell lines and an increased pathogenic potential in various animal models in combination with an even faster growth in vivo (not in vitro).

**Conclusion:**

Molecular, genomic and phenotypic analysis of representative isolates of a major clone of *E. faecalis* MLST ST40 revealed new insights into the microbiology of a commensal bacterium which can turn into a conditional pathogen.

**Electronic supplementary material:**

The online version of this article (doi:10.1186/s12864-015-1367-x) contains supplementary material, which is available to authorized users.

## Background

Enterococci constitute an integral part of the intestinal flora of many invertebrates, birds and mammals including humans. Recent studies on livestock animals have improved our understanding of the role enterococci play as important intestinal colonisers by supporting intestinal, microbial homoeostasis, stimulating immune modulation and thus preventing infections with pathogenic bacteria and viruses [1-3]. Commercial probiotic mixtures of *Enterococcus faecalis* are sold with a supposed supportive role in anti-inflammation and prevention of allergic reactions. The genome sequence of one of these probiotic strains called Symbioflor 1 has been released very recently [4]. Enterococci have a supportive activity in food fermentation and preservation, because of their production of secondary metabolites and bacteriocins [5]. On the other hand, enterococci, in particular *E. faecalis* and *E. faecium*, are important conditional pathogens. *E. faecalis* has been attributed to various kinds of infections in humans in hospitals and in the community such as urinary tract infections, bacteraemia and/or endocarditis [6-9].^a^ In animals it is a common cause of mastitis in cattle [10], and of urinary tract infections in dogs and cats.

In *E. faecalis*, a number of ‘classical’ virulence factors are essential for different kinds of and the course of infections. A cytolysin executes cytolytic, haemolytic and bacteriocinogenic activities capable of lysing prokaryotic and eukaryotic cells [11]. The expression of the two-component cytolysin is highly regulated and controlled by quorum-sensing [12]. Relevant genes are arranged in an operon structure which is encoded on a pathogenicity island (PAI) and/or a pheromone response plasmid of the pAD1 type [13,14]. The association of cytolysin expression and increased toxicity of *E. faecalis* infections has been shown in various animal models, as well as in outcome-oriented clinical studies [15,16]. Other well-described virulence factors such as gelatinase and serine protease have been singly shown to contribute to tissue invasion and translocation and thus to pathogenicity of *E. faecalis* infections in general [9,17-19]. Corresponding genes *gelE-sprE* are genetically linked, co-regulated by *fsr* and co-transcribed [18,20,21].

Examples of *E. faecalis* surface-exposed virulence markers are capsular polysaccharides, glycolipids, surface-exposed LPxTG-type proteins, such as microbial surface components recognising adhesive matrix molecules (MSCRAMMs), like the collagen-binding protein Ace, pili called Ebp, and aggregation substances [8,22-26]. Surface protein-mediated adherence to host tissues leads to the first steps of enterococcal colonisation and subsequent biofilm formation, inflammation and infection.

The enterococcal surface protein Esp is known to be involved in biofilm production and surface attachment [27-29]. The corresponding *esp* gene is part of the *E. faecalis* and *E. faecium* PAI which shows different sizes and compositions in both species [14,30]. The *E. faecalis* PAI in the reference isolate MMH594 is 153 kb long and part of the chromosome [14]. In *E. faecium* only isolates from clinical infections contain the PAI, whereas in *E. faecalis* commensal (human, animal) and environmental strains may also contain it [31,32]. Supposed virulence genes of *E. faecalis* isolates as described above are also distributed among commensal and environmental isolates [33-35]. The exact composition of the *E. faecalis* PAI varies in different strains. Differences appear in the presence and absence of the six blocks described for the original PAI of MMH594 which are flanked by mobile genetic elements [32]. Parts of the PAI including the 5′ encoded aggregation substance may be derived from the integration of plasmid fragments in the PAI. Aggregation substance is also an integral part of various types of *E. faecalis* pheromone plasmids of the pAD1-, pCF10- and pAM373-types [13,36]. The aggregation substance is essential in linking donor and recipient cells in pheromone-induced mating processes but also supports attachment to eukaryotic surfaces and intracellular routes of immune evasion [37,38].

The enterococcal capsule locus (*cps*) consists of 11 known open reading frames, namely *cpsA*-*K*. There are three known capsule operon polymorphisms: (1) *cps* type 2, which includes all 11 genes (e.g. V583); (2) *cps* type 5, which includes all genes except for *cpsF* (like strains of the CC9); and (3) *cps* type 1, where only *cpsA* and *cpsB* are present (e.g. OG1RF) [24,39].

Horizontal gene transfer (HGT) is supposed to play a key role in shaping enterococcal genomes, bacterial functionality and *E. faecalis* phylogeny [40,41]. This is exemplified by (1) the high number of mobile genetic elements in some sequenced *E. faecalis* genomes, such as V583 summing up to >25% and (2) recent phylogenetic analyses based on whole genome data [41,42]. Various integrative and conjugative elements (ICE) are known in *Enterococcus*, such as the conjugative tetracycline resistance transposons of the Tn*916*-type and the aforementioned pheromone response plasmids, which allow very efficient gene transfer rates of up to 10e-1 per donor cell. In addition, broad host range plasmids of the Inc*18*-type are also prevalent among *E. faecalis* which became prominent when transmitting *vanA*-type vancomycin resistance from *E. faecalis* to MRSA [43,44]. ICE can also mobilise other parts of the genomes when transferred as shown recently [45,46]. In *E. faecium* genome remodelling associated with acquisition and loss of mobile genetic elements has contributed to ecological niche separation with respect to domestic animals and to the evolution of a hospital-associated subpopulation as a cause of infections in immunocompromised humans; within ecological niches and/or distinct clonal lineages or complexes the level of genomic variance is minor [47-49].

Data from DNA sequence-based typing of *E. faecalis* assessed by MLST suggest that a number of prominent clonal complexes which are common among animal and human isolates such as CC2, CC16, and CC40, do not suggest any kind of host specificity and also less abundant clonal lineages were reported from urinary tract infections in humans and chickens [50]. A phylogenetic analysis with respect to ecological separation and host specificity had not been performed for *E. faecalis* at the time when we initiated our study. Typing by means of MLST of 386 *E. faecalis* isolates of a European collection revealed that specific types determined by MLST analyses and subsequent eBURST clustering mainly belong to six clonal complexes (CC2, CC16, CC21, CC30, CC40 and CC87) which play a predominant role in the spread of antimicrobial resistance in hospitals and contribute to higher resistance rates in some countries [51]. In order to elucidate further the phylogenetic structure of a prominent MLST type and display possible routes of niche adaptation we focused on the most prevalent clonal type ST40 [39]. We collected and typed 42 ST40 isolates from worldwide and divergent ecological sources by phenotypic and molecular means and characterised a subset of them by whole genome sequence comparisons, functional assays including BIOLOG analyses and animal experiments of colonisation and pathogenicity.

## Methods

### Strain collection

Forty-two isolates of *E. faecalis* MLST type ST40 were collected from colonisation studies in different animals, people in the community and hospital patients, from different kinds of infections in humans and animals and from food. They originated from different countries and two continents and spanned a range of >50 years. A detailed list of the ST40 strains with the corresponding background information is described in Additional file 1: Table S1; summarised information is given in Table 1. We did not use or receive original clinical samples but only bacterial isolates. Bacteria and plasmids used for reference purposes are given in Table 2.

**Table 1:** ***E. faecalis*** **ST40 strain collection used in this study**

***Isolate***	***Country (City)***	***Year***	***Origin***
UW1833 (U 09508/98)*	D (Berlin)	1998	H, U
UW5212	D (Cologne)	2004	H, U
UW5744	D (Cologne)	2004	H, U
UW6530	D (Augsburg)	2006	H, U
UW6756	D (Leezen)	2006	H, U
UW6724 (Ba7514)*	D (Wernigerode)	2006	H, C
UW6727 (Ba7517)*	D (Wernigerode)	2006	H, C
UW7775	D (Heidelberg)	2004	H, C
UW7776	D (Heidelberg)	2004	H, C
UW7777 (AK-EF 29)*	D (Heidelberg)	2004	H, C
UW7778	D (Heidelberg)	2004	H, C
UW7779 (AK-EF 92)*	D (Heidelberg)	2004	H, C
UW2860 (3803)*	D (Gera)	2000	H, B
UW2861	D (Gera)	2000	H, B
UW4340	D (Berlin)	2003	H, B
UW4889	D (Augsburg)	2004	H, B
UW5209	D (Augsburg)	2004	H, B
UW5213	D (Augsburg)	2004	H, B
UW5345	D (Augsburg)	2004	H, B
UW6149 (AB 5093–231)	D (Augsburg)	2005	H, B
UW7800 (ATCC 27275)	Unknown	≤1962	Unknown
UW7801 (ATCC 27959)*	USA (Iowa)	≤1975	A^1^, M
UW7729 (LMGT 2333)*	IS (Reijkjavik)	1990	A^2^, C
UW7730 (LMGT 3209)	GR (Athens)	<2004	F
UW7709 (5)*	DK	1997	H, E
UW7710 (6)	DK	>2000	H, U
UW7742 (D1)*	DK	2001	A^3^, C
UW7743 (D27)	DK	2001	A^3^, C
UW7744 (D32)^▲^	DK	2001	A^3^, C
UW7745 (D37)	DK	2001	A^3^, C
UW7761 (DQ213/C003-e)*	Cuba (Havana)	2008	H, B
UW7753 (HC 24)*	ESP (Madrid)	2001	H, B
UW7780 (402/96)*	PL (Warsaw)	1996	H, C
UW7781	PL (Warsaw)	1996	H, PF
UW7782	PL (Warsaw)	1996	H, U
UW7784	PL (Grajewo)	1999	H, U
UW7785	PL (Zawiercie)	2000	H, W
UW7787	PL (Zamość)	2002	H, CSF
UW7788	PL (Bytom)	2003	H, B
UW7789	PL (Biała Podlaska)	2004	H, U
UW7790	PL (Wołomin)	2007	H, C
UW7791	PL (Maków Maz.)	2007	H, C

*E. faecalis* strain collection comprises 42 ST40 strains. * draft genomes; ^▲^ ST40 complete reference genome; A, animal (^1^ cow, ^2^ fish, ^3^ pig); B, blood culture; C, colonizer; CSF, cerebrospinal fluid; E, endocarditis; F, food (cheese); H, human; M, bovine mastitis; PF, peritoneal fluid; U, urine; W, wound.

**Table 2:** **Bacterial strains and plasmids used as references**

***Strain***	***MLST***	***Resistance***	***Plasmids***	***Description***	***References***
***E. faecalis:***					
V583	CC2 (ST6)	VAN, ERY, STR	pTEF1, pTEF2 and pTEF3	ATCC700802; first sequenced *E. faecalis* genome	[42]
MMH594	CC2 (ST6)			Contains the first identified complete PAI	[14]
OG1RF	ST1	RAM, FUS	Plasmid-free	ATCC 47077; mutant of OG1	[65]
***E. faecium:***					
64/3	ST21	RAM, FUS	None	Commensal isolate; standard recipient	[52]
***Staphylococcus aureus:***					
NCTC8325				Size marker in PFGE	
**Plasmids:**					
			pAD1	Isolated from *E. faecalis* OG1::pAD1	[13]
		TET	pCF10	Isolated from *E. faecalis* OG1::pCF10	[36]
		TET, CMP, ERY, STR	pRE25	Isolated from *E. faecalis* RE25::pRE25	[101]

CC, clonal complex; ST, sequence type; only antibiotic resistances relevant for this study are presented: CMP, chloramphenicol; ERY, erythromycin; FUS, fusidic acid; RAM, rifampicin; STR, streptomycin (high-level); TET, tetracycline; VAN, vancomycin.

### Antibiotic resistance

Antibiotic susceptibilities were determined by broth microdilution and according to EUCAST guidelines using clinical breakpoints or epidemiological cut-off values (http://www.eucast.org/clinical_breakpoints/).

### Cytolysin/haemolysin and gelatinase assays

As previously described [45], in vitro β-haemolytic activity was qualitatively analysed by the use of MH agar plates containing 5% human blood in combination with 1% glucose (Merck KGaA, Darmstadt, Germany) and 0.03% L-arginine (Sigma-Aldrich GmbH, St Louis, USA). In vitro gelatinase expression, resulting in hydrolysis of gelatin, was determined using Todd-Hewitt agar plates (OXOID GmbH, Wessel, Germany) containing 3% gelatin (Becton, Dickinson & Co., New Jersey, USA) [45].

### Isolation of the whole genome DNA

Genomic DNA was isolated by using column-based methods (Qiagen GmbH, Hilden, Germany). The final DNA concentration was determined using Quant-iT® PicoGreen® dsDNA Quantitation Reagent (Invitrogen®- Molecular Probes Inc., Paisley, UK) following the instructions of the manufacturer and the quality of the DNA preparation was visually inspected in agarose gels.

### Plasmid DNA isolation

Preparation of plasmid DNA was done by using a phenol/chloroform-based extraction method as described recently [52].

### PCR and long template PCR

Standard PCR reactions were performed using PCR Master mix (Thermo Fischer Scientific Inc., Waltham, USA) in accordance to the manufacturer’s instructions. Long template PCR using the Expand Long Template PCR kit (Roche Biochemicals, Mannheim, Germany) was performed to amplify the integration site of a PAI, as well as to analyse its structure according to the reference structure in strain MMH594 and as described recently [45]. All primers used are listed in Additional file 2: Table S2.

### Multilocus sequence typing (MLST)

MLST was performed according to the *E. faecalis* MLST scheme by amplifying seven housekeeping genes [53]. MLST sequence types (ST) were generated by using the MLST website (http://efaecalis.mlst.net/; last access: 03.03.2015).

### PFGE and Southern hybridisation

*Sma*I macrorestriction and subsequent PFGE analysis were done as described previously [10]. CHEFF III apparatus (BIO-RAD, Munich, Germany) was used. For plasmid PFGE an S1 nuclease treatment procedure was used as described recently [54]. As an external size standard, *Sma*I-digested *S. aureus* NCTC8325 strain was used to calculate the fragment sizes with BioNumerics version 6.0 software (Applied Maths, Sint-Martens-Latem, Belgium).

Southern hybridisations and immunological detection were done with DIG High Prime system kits and CDP-Star detection (Roche Biochemicals, Mannheim, Germany) following the manufacturer’s recommendations. In the case of plasmid classification, labelled probes were generated by using DIG-labelled dUTP and primers repCF10-1/2 and repRE25-1/2 for plasmid replicon classification (Additional file 2: Table S2).

### Sequencing by Sanger ABI Big Dye technology

PCR products were sequenced by a cycle sequencing approach according to the recommendations of Applied Biosystems (Darmstadt, Germany). Sequencing amplicons were determined at the central sequencing facility of the RKI. Sanger reads were analysed by Lasergene 8 (DNASTAR, Madison, USA) or DS Gene software packages (Accelrys, Inc., San Diego, USA).

### Roche/454 FLX genomic pyrosequencing

Fifteen representative isolates were selected for de novo sequencing by Roche/GS-FLX 454 technology. To generate a completely closed, circular ST40 reference genome the porcine strain D32 was used for combined long and short template paired end library 454 sequencing at MWG Biotech (Ebersberg, Germany) as described briefly in [55]. Genomic contigs were generated and assembled by using the Newbler assembler software (Roche Diagnostics, Basel, Switzerland).

### Illumina/Solexa sequencing and hybrid assembly

To improve the accuracy of the sequencing data, genomes of the remaining 14 *E. faecalis* strains (without D32 ([55]) were additionally sequenced by using v2 chemistry and Illumina’s Genome Analyzer IIx (Nextera DNA Sample Preparation Kit, Illumina, San Diego, USA). De novo sequences, generated by both Roche/454 FLX pyrosequencing and Illumina/Solexa sequencing, were assembled in a single approach (hybrid assembly) by using Mira assembler software (Sourceforge/Dice Holdings, Inc., N.Y., USA).

### Genomic comparisons and phylogenetic analyses

Generating a reference genome of isolate D32 was described previously [55]. Annotation was done with RAST and GenDB [55]. Circular maps visualising the *E. faecalis* ST40 genome comparison against the D32 reference genome were generated by BRIG [56]. Alignments for the phylogenetic tree were calculated with Mugsy [57] and only the aligned regions present in all analysed strains were extracted (‘core genome’). These regions were concatenated and positions with gaps removed [58]. The resulting core alignment was used to infer a Maximum Likelihood tree with RAxML [59]. The GTRGAMMA model for nucleotide substitution and rate heterogeneity was utilised, and bootstrap support values of 1000 replicates were shown at the nodes. Additional phylogenetic analyses of the 15 *E. faecalis* ST40 isolates were performed as follows: Solexa single reads were mapped onto the reference sequence of *E. faecalis* D32 by utilising a pipeline based on bwa version: 0.7.3a (http://sourceforge.net/projects/bio-bwa/files/) for mapping, SAM tools 0.1.18 (http://samtools.sourceforge.net/) for SAM/BAM file handling and VarScan v2.3 (http://varscan.sourceforge.net/index.html) for variant calling. In this way consensus sequences aligned to the reference sequence were generated and all variable sites were extracted. To exclude SNPs resulting from recombination the distances between SNP positions were analysed to find pairs within a certain range (exclusion distance ≤300 bp). Subsequently, a total of 1481 variable positions were used for tree calculations by the PhyML algorithm with a bootstrap of 1000. The genome sequence of *E. faecalis* D32 is deposited at GenBank numbers NC_018221-018223. All other scaffolded *E. faecalis* genome sequences are deposited at JGI’s IMG/ER website (https://img.jgi.doe.gov/cgi-bin/er/main.cgi) under the accession numbers 22305 – 22317 (see also Additional file 1: Table S1).

### Biolog phenotyping microarrays

Biolog Phenotyping Microarrays (PM01 and PM02A MicroPlate™; BIOLOG, Hayward, USA) were used to analyse the metabolic phenotype with focus on utilisation of different carbon sources under aerobic conditions. Briefly, bacteria were incubated on fresh MH agar plates (OXOID GmbH, Wessel, Germany) at 37°C for 24 h. With a sterile swab, bacteria were transferred into a sterile tube containing 10 mL IF-0a medium (1.2x) (BIOLOG), and cell suspension was adjusted to 81% transmittance by using the Biolog turbidimeter (BIOLOG). In line with the manufacturer’s recommendations, 20 mL of the IF-0a Base (1.2x) was mixed with 0.24 mL Redox Dye Mix D (100x) (both BIOLOG) and 2.0 mL of a 12x additive solution, containing 24 mM MgCl_2_ × 6H_2_O (CARL ROTH GmbH & Co. KG, Karlsruhe, Germany) and 12 mM CaCl_2_ × 2 H_2_O (CARL ROTH GmbH & Co. KG, Karlsruhe, Germany). Finally, 1.76 mL of cell suspension was added. 100 μL of the final suspension was added to each well of the 96-well Biolog PM01 and PM02A plates. The panels were placed in the OmniLog instrument (BIOLOG) and incubated at 37°C for 72 h, whereby the utilisation of the carbon sources was measured spectrophotometrically and recorded every 15 min over the whole incubation period. All assays were repeated for at least three times on different days. Data sets were evaluated by the OmniLog Kinetic and Parametric analysis software version 2005 (BIOLOG Life Science Institute, Bremen, Germany). The area under the kinetic curve (area values) was used to compare the utilisation of different carbon sources between the strains. Calculation of the arithmetic averages with the corresponding standard deviations was done with Microsoft Excel. Colour codes, used in Additional file 2: Table S6a and b, were chosen according to Gripp and colleagues [60].

### Growth kinetics

To determine the bacterial growth rates, overnight cultures were diluted 1:50 in TS broth and grown at 37°C with shaking. Optical density at 600 nm was measured at different time points and the corresponding CFU calculated by plating serial dilutions of the cultures onto LB agar plates in duplicate.

### Biofilm plate assay

The ability of selected *E. faecalis* strains to produce biofilm on flat bottom polystyrene microtiter plates (Greiner Bio-one, Germany and Corning Inc., New York, USA) was tested by following the methodology previously described [45]. Biofilm plate assay for each of the strains was done in triplicate and repeated twice. Statistical comparisons were done by unpaired two-tailed t-tests using a GraphPad Prism 5.01 software package (GraphPad Software, Inc., La Jolla, USA). A *P*-value of <0.05 was considered statistically significant.

### Adherence assays

Adherence to human epithelial colorectal adenocarcinoma Caco-2 cells was investigated using a protocol previously described [22,23] with slight modifications. Caco-2 cells between the 15th and 25th passages were cultivated in 24-well plates to a density of 1 × 10^5^ cells/well for 13 to 15 days (confluent monolayer). The monolayers were incubated with a bacterial cell to epithelial cell ratio of 100:1, as well as 1000:1 for 2 h. After infection of the monolayer, epithelial cells were washed five times with phosphate saline buffer (PBS, Biochrom AG) and lysed with 0.25% Triton-X100 (Sigma) at 37°C for 15 minutes. To determine quantitatively the number of attached bacterial cells, lysates were diluted in TSB (tryptic soy broth) and plated onto TSA plates. Statistical comparisons of cfu numbers were done by unpaired two-tailed t-tests using the GraphPad Prism 5.01 software package (GraphPad Software, Inc., La Jolla, USA).

### Animal models

Different animal models were used for a comparative assessment of the pathogenic potential of the selected enterococcal strains D32 and UW7709. Also, *E. faecalis* strains V583 and OG1RF, as well as *E. faecium* 64/3, served as controls.

### Assay of G. mellonella infection

The insect larva *G. mellonella* is an alternative model for studying bacteria-host interactions, showing a complex immune reaction consisting of both cellular and humoral responses. According to previously described protocols [61,62], assays were done with some modifications. Some 100 μL of TSB overnight culture was added to 5 mL of fresh TSB medium (Becton, Dickinson & Co., Heidelberg, Germany) and cultured at 37°C for 3 h. After centrifugation for 5 min at 8,000 rpm, cell pellets were resuspended in 1 mL sterile PBS. Cell concentration was photometrically measured and the cell density of the inoculum was adjusted to 10^7^ cells/500 μL. Groups of 15 *G. mellonella* larvae (www.reptilienkosmos.de) with a weight of about 200 mg were separated. Then, 5 μL of the bacterial inoculum was microinjected at the base of the last proleg, corresponding to an infective dose of 10^5^ CFU/larvae. A control group of larvae was infected with PBS only. The real infective dose was determined by serial dilution, plated on PBS agar plates. Groups of infected larvae were kept per Petri dish at 37°C and the number of dead larvae was counted after 18, 24, 42, 48, 66 and 70 h. This approach was repeated at least three times for each of the selected strains. Using the nominal values of survival and death, the diagram, presenting the death rates, was calculated by Kaplan-Meier plot method in GraphPad Prism 5.01 software version (GraphPad Software, Inc., La Jolla, USA). Statistical significance (p < 0.05) was determined by the Log-rank (Mantel-Cox) test.

### Murine bacteraemia model

The murine bacteraemia model was used to evaluate the pathogenic potential of the selected *E. faecalis* strains D32 and UW7709 by analysis of bacterial growth in blood and murine organs (liver, kidney and spleen) following a methodology described previously [23,63]. In brief, eight female 6-8-week-old BALB/c mice (Charles River Laboratories Internations, Inc., Wilmington, USA) were inoculated intravenously in the tail vein with 10^8^ and 5×10^8^ CFU respectively and bacterial growth was analysed after 48 h post-infection. Statistical comparisons were done by Mann–Whitney tests (non-parametric data) using the GraphPad Prism 5.01 software package (GraphPad Software, Inc., La Jolla, USA).

Accession numbers. The full genome sequence of E. faecalis D32 is deposited at GenBank numbers NC018221-018223. All other scaffolded E. faecalis genome sequences are deposited at JGI’s IMG/ER website (https://img.jgi.doe.gov/cgi-bin/er/main.cgi) under the accession numbers 22305 – 22317 (see also Additional file 1: Table S1).

## Results

### Pre-characterisation of the ST40 strain collection

**Figure 1:**
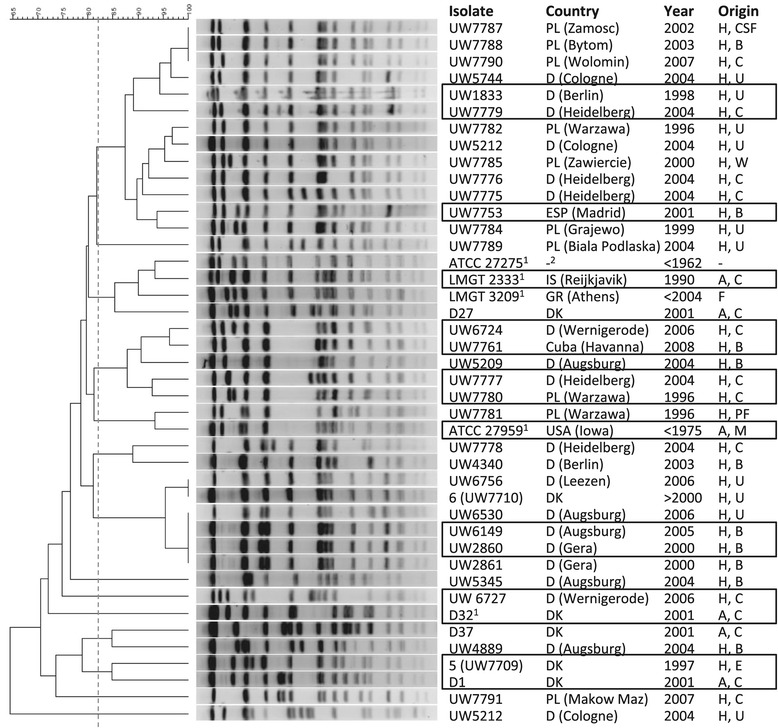
***Sma*** **I macrorestriction patterns in PFGE of 42** ***E. faecalis*** **ST40 isolates.** The phylogenetic analysis was generated with BioNumerics 6.0. The scale bar represents the level of similiarity in % using default settings (tolerance 1.0; optimization 0.5). The dotted vertical line at 82% similarity delineates the breakpoint for defining related fragment patterns/strains according to an international agreement. Certain sub-clusters indicated a comparably high level of clonal relatedness. Sequenced isolates are marked with a black square. Legend: H, human; A, animal; F, food; ^1^ reference strain; B, blood culture; CSF, cerebrospinal fluid; C, colonizer; E, endocarditis; F, food; M, bovine mastitis; PF, peritoneal fluid; U, urine; W, wound. Origin: D, Germany; DK, Denmark; PL, Poland; IS, Island; GR, Greece; ESP, Spain.

The ST40 strains were compared for specific phenotypic and genotypic characteristics in order to select representative isolates for genome sequencing. Strains were typed by *Sma*I macrorestriction in PFGE and sub-clusters of closely related strains were identified which were grouped independently of their geographical and temporal origin or clinical/non-clinical context (Figure 1). Distribution of phenotypic antibiotic susceptibilities was completed by determination of the corresponding genes by PCR (Table 3; Additional file 1: Table S1). Results were mainly congruent. Two discrepancies were detected; one isolate was *aadE-*positive but not streptomycin-high-level resistant and another isolate was *aac6’-aph2”*-negative but gentamicin-resistant. Presence and expression of putative virulence-associated genes encoded within the *E. faecalis* PAI and/or on the chromosome were investigated by PCR and partly confirmed by phenotypic in vitro assays (Table 4). The PAI-associated aggregation substance *asc-10* gene was found in 16.7% of the isolates. Prevalence of the cytolysin (*cy*l) operon in 33.3% of the strains was associated with the evidence of β-haemolysis in vitro. The enterococcal surface protein gene *esp* was detected in 78.6% of the ST40 strains. All isolates harboured the *gelE* (gelatinase) and *fsr* (major accessory gene regulator) genes and showed in vitro gelatinase expression (Table 4; Additional file 1: Table S1).

**Table 3:** **Distribution of antibiotic resistances in ST40** ***E. faecalis*** **isolates**

***Antibiotic resistance***	***Phenotype [%]***	***Resistance gene***	***Genotype [%]***
Vancomycin (VanA-type)	2.4 (1/42)	*vanA*	2.4 (1/42)
Erythromycin	21.4 (9/42)	*erm*(B)	21.4 (9/42)
Tetracycline	81.0 (34/42)	*tet*(M)	81.0 (34/42)
Streptomycin (high-level)	31.0 (13/42)	*aadE*	33.3 (14/42)
Gentamicin (high-level)	2.4 (1/42)	*aac6′-aph2”*	0 (0/42)

Broth microdilution assay was used to determine phenotypic antibiotic susceptibilities. Resistance genes were determined by PCR.

**Table 4:** **Presence and expression of putative virulence factors in ST40** ***E. faecalis*** **isolates**

***Putative virulence factor***	***Putative function***	***% of positive isolates***	***Comment***
*Pathogenicity island*			
*asc-10*	Aggregation substance	16.7 (7/42)	
*cylM*	Cytolysin subunit modifier	33.3 (14/42)	Expression of β-hemolysis *in vitro*
*esp*	Enterococcal surface protein	78.6 (33/42)	
*xyl kinase*	Xylose kinase	4.8 (2/42)	
*gls24-like*	General stress protein	0 (0/42)	
*Other*			
*gelE*	Gelatinase/coccolysin	100 (42/42)	Expression of gelatinase *in vitro*
*fsrB*	Accessory gene regulator B	100 (42/42)	
*cps* type 1	Capsular polysaccharide	100 (42/42)	

Presence of putative virulence-associated genes (in italics) was examined by PCR. Numbers in bold represent percentage of positive tested isolates, calculated from the number of the positive genotypes versus the whole strain collection (in parentheses).

The enterococcal capsule locus (*cps*) consists of 11 known open reading frames, namely *cpsA*-*K*. Capsule locus type 1 (*cpsA-cpsB*) was verified by PCR for all ST40 isolates.

We also tested the ability of the ST40 strains to form biofilms on polystyrene plates. Biofilm formation was independent of the presence of putative biofilm-enhancing genes like *esp* and *asc-10*, and was also inhomogeneous between closely related strains (Additional file 3: Figure S1).

Results of plasmid isolation in combination with S1 nuclease PFGE indicated diversity in plasmid content, varying in size and quantity (none to two plasmids) and plasmid *rep* type (see “Comparative analysis of the *E. faecalis* ST40 genomes” and Additional file 4: Figure S2).

### Genome sequencing

On the basis of previous characterisations, a subset of 15 strains (Figure 1), representing the diversity of the ST40 collection and some pairs of related isolates, was sequenced de novo by Roche GS FLX 454 technology. Genomic contigs were generated and assembled by using the Newbler assembler software (Additional file 2: Table S3). Although in principle suitable for de novo genome assemblies, classical 454 sequencing revealed only two strains (D32 and UW7709) with fewer than 100 genomic contigs. The calculated genome size varied from 2.8 to 3.3 Mbp. For the majority of the genomes coverage was unsatisfactory (<20x). To improve the accuracy and overall quality of the sequencing data, a subsequent workflow was pursued including: (1) establishing a template for genomic mapping within ST40 by generating a completely closed chromosome of a reference isolate using long paired end 8 kbp libraries and 454 sequencing as well as classical Sanger sequencing to correct for InDel errors; and (2) additional sequencing of the other 14 *E. faecalis* strains by Solexa technology (see also Methods and ff).

### Generating a reference genome of *E. faecalis* D32

Key parameters of the sequencing, assembly and annotation strategy were described recently as well as standard information regarding numbers of ribosomal genes, coding sequences, etc. [55]. Additionally to what we have elucidated in this previous announcement, subsequently performed S1 nuclease PFGE analyses revealed a single ca. 75 kb plasmid in strain D32. Regarding the two supposedly assembled plasmid contigs of 62 and 13 kb and the fact that the former plasmid EFD32pB did not show any homology with a replicase gene, we postulated an assembly error and suggested the two contigs merge into one single plasmid sequence demonstrating rep1 type.

**Figure 2:**
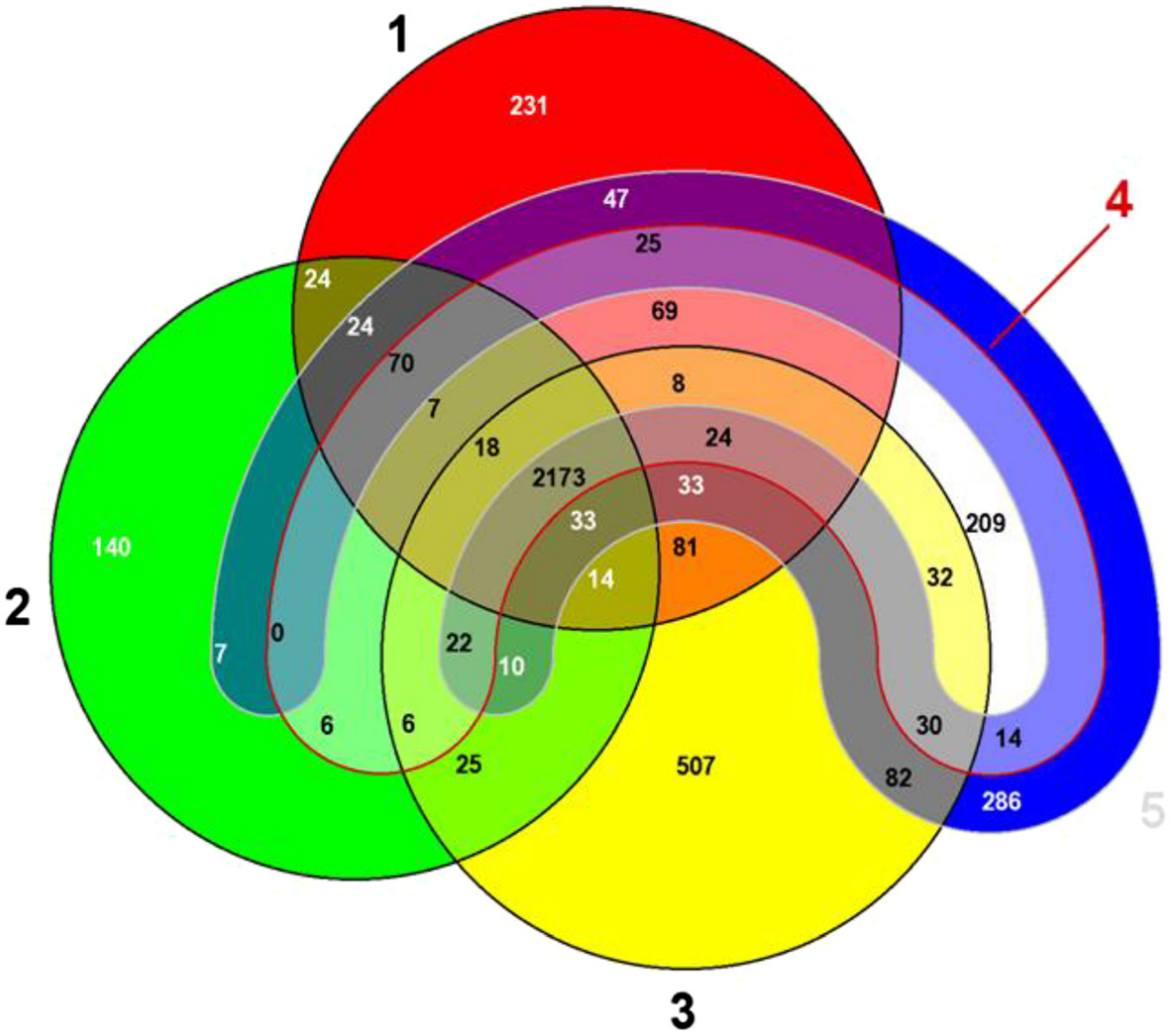
**Comparative analysis of the finished and publicly available** ***E. faecalis*** **genomes.** EDGAR generated Venn diagram facilitates visualizing core and strain-specific (“unique”) genes. This comparative analysis only exploits CDS of the chromosomes without considering of plasmid genes, whereby all strains shared 2173 CDS; 1, *E. faecalis* strain 62 (CP002491); 2, *E. faecalis* strain OG1RF (CP002621); 3, *E. faecalis* V583 (NC_004668); 4, *E. faecalis* probiotic strain Symbioflor 1 Clone DSM 16431 (NC_019770); 5, *E. faecalis* strain D32 (CP003726).

We used a Venn diagram presentation generated by the web application EDGAR to illustrate homologies and differences between the finished and publicly available *E. faecalis* genomes. It illustrated a common gene pool of 2173 CDS, present in all of the finished *E. faecalis* genomes (Figure 2). This analysis also revealed that the number of unique CDS of the clinical strain V583 was approximately twice that of the counts of the commensal isolates 62 and our ST40 isolate D32 as well as the probiotic Symbioflor 1 strain. As a derivate of the commensal isolate, the OG1RF strain carried the minimal number of 140 unique CDS. The commensal strains 62 and D32 both shared significantly more CDS with the V583 chromosome, whereas the Symbioflor 1 and OG1RF chromosomes overlapped less with the V583 core genome.

### Comparative analysis of the *E. faecalis* ST40 genomes

**Figure 3:**
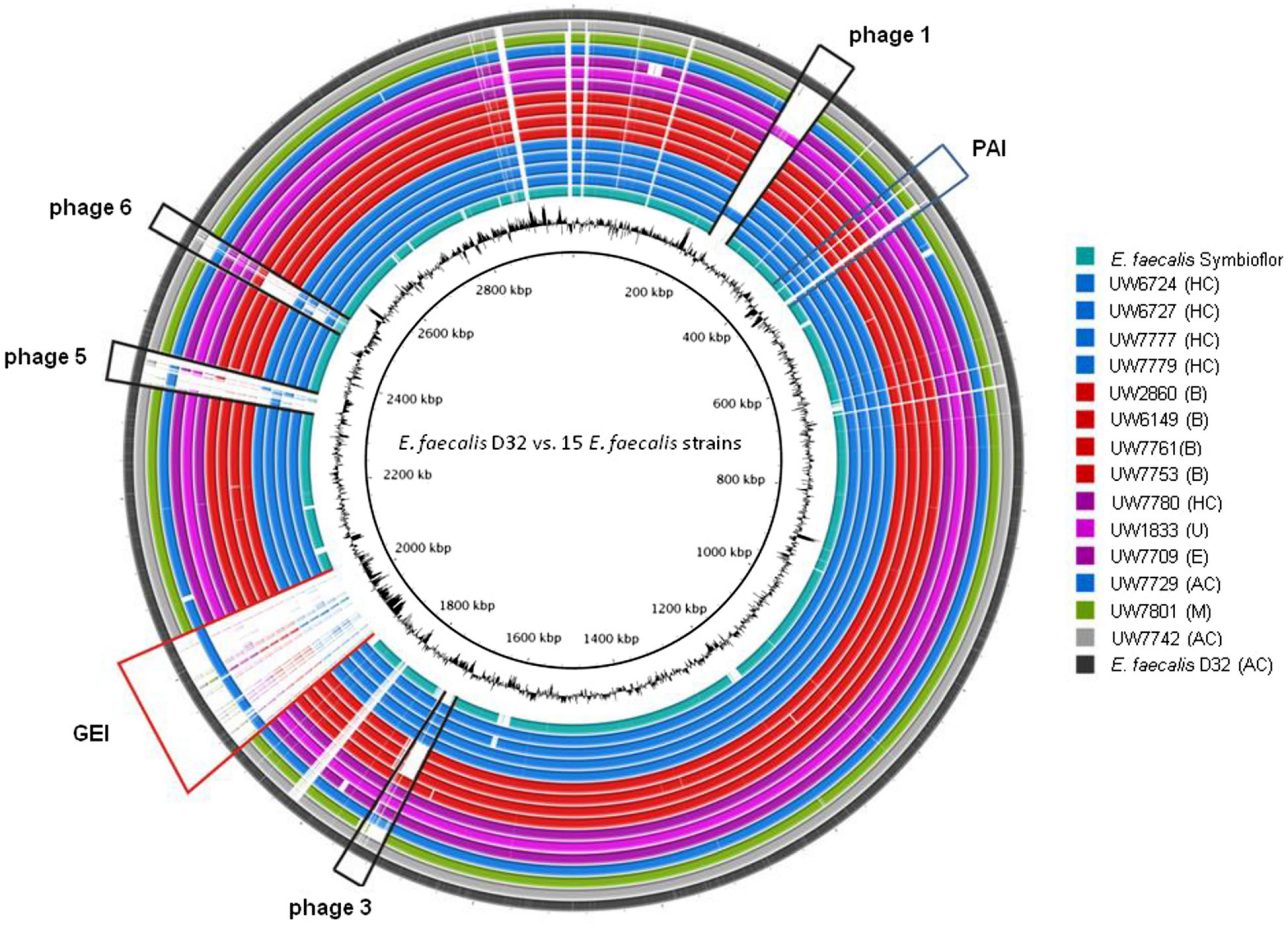
***E. faecalis*** **ST40 genome comparison against the D32 reference genome.** Generated by BRIG (http://brig.sourceforge.net/ [last access 16.07.2014] [56]), the circular map illustrates the whole genome comparison of D32 against the other 14 sequenced ST40 isolates and the probiotic isolate Symbioflor 1 Clone DSM 16431. The outer cycle (dark grey) represents the complete genome of the reference strain D32. The shade of color is geared to similarities in origin of the strains (green: isolate from bovine mastitis; blue: animal and human commensals; violet: isolates from human infections; red: human blood culture isolates; turquoise: strain Symbiolfor 1). The inner cycle illustrates the GC content of D32. Location of the PAI is illustrated by a blue colored box, while the red box indicates the presence of an uncharacterized and large genomic island (GI; 138 kb). Additionally, black labels highlighted four identified prophages of D32; A, animal; B, blood culture; C, colonizer; E, endocarditis; H, human; M, bovine mastitis; U, urine.

De novo sequences, generated by both 454 pyrosequencing and Illumina/Solexa sequencing, were hybrid assembled. This combined sequencing and assembly approach resulted in satisfactory analysis parameters since it improved overall coverage and read length, slightly increased the genome size (2.928 - 3.33 Mbp) and reduced the number of genomic contigs (<100; Table 5). Mapping of the 14 ST40 draft genomes against the D32 reference genome suggested a high level of genomic similarity irrespective of the geographical, host, temporal or clinical/non-clinical origin of the isolates (Figure 3). Differences between the strains were minor and corresponded to (1) a different composition of the *E. faecalis* PAI, (2) differences in phage content and (3) a putative genomic island first described in D32 which is absent in all other ST40 strains except for another animal isolate, UW7729. This genomic island was located at a putative hot spot for integration in the *E. faecalis* chromosome, since strain V583 contained the *vanB* operon at this site whereas OG1RF harboured the myo-inositol operon (which D32 lacks).

**Table 5:** **Quality report of hybrid assemblies considering of 454 and Solexa sequencing data**

**Isolate**	**No. of assembled reads**	**Coverage (454) [n-fold]**	**Coverage(Solexa) [n-fold]**	**No. scaffolds**	**Genome size [bp]**	**GC content [%]**
UW6149	5,060,490	12.78	166.36	72	3,011,563	37.46
UW2860	5,318,329	14.68	176.92	74	3,003,615	37.49
UW6724	1,232,615	9.91	39.94	38	3,085,224	37.33
UW7761	2,025,698	12.71	81.94	26	2,939,826	37.53
UW7777	1,504,959	24.05	45.28	24	2,999,678	37.43
UW7780	1,108,686	10.91	37.28	38	3,115,640	37.31
UW7753	1,424,574	11.14	39.48	67	3,119,011	37.09
UW1833	3,127,985	14.89	100.92	66	3,243,986	37.05
UW7779	1,412,336	18.92	37.1	45	3,070,536	37.20
UW7729	972,823	9.01	28.64	75	3,094,285	37.19
UW7801	1,478,988	10.79	56.72	26	2,933,628	37.52
UW6727	1,442,566	12.5	45.58	34	3,330,760	36.91
UW7709	2,403,224	12.98	90.18	36	2,928,951	37.30
UW7742	3,271,151	18.06	101.23	64	3,054,136	37.17

Genomes of 14 selected ST40 isolates were sequenced by using Solexa technology. Reads of 454 and Solexa sequencing were hybrid assembled by using Mira assembler software and resulted in an increased coverage in combination with a reduction of the number of large contigs.

**Figure 4:**
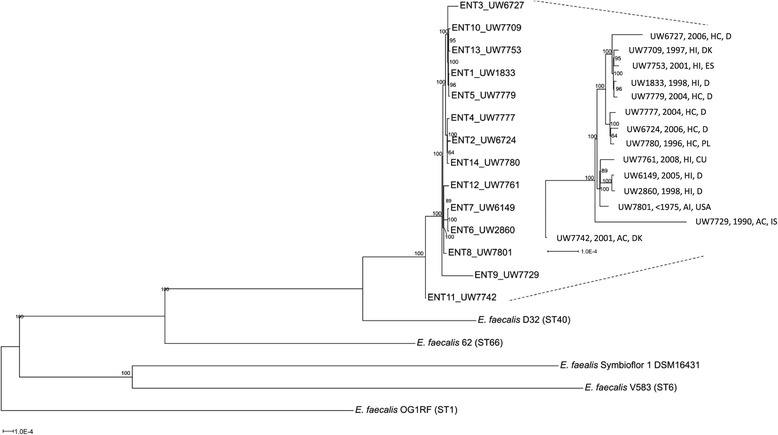
**Phylogenetic relationship among selected** ***E. faecalis*** **strains based on whole genome alignments.** The alignment was calculated with Mugsy (http://mugsy.sourceforge.net/ [last access 16.07.2014] [57]) and only aligned regions present in all analyzed strains were extracted. These regions were concatenated and positions with gaps removed. The resulting core alignment was used to infer a Maximum Likelihood tree with RAxML. The GTRGAMMA model for nucleotide substitution and rate heterogeneity was utilized, bootstrap support values of 1000 replicates are shown at the nodes. Names of the ST40 isolates and their origin are indicated at the end of the branches. The highly related ST40 isolates were further zoomed in exemplified by the dotted line and the different scale bar. Metadata are given as follows: Strain no., year of isolation, origin, country: AC, animal colonizer; AI, animal infection; HC, human colonizer; HI, human infection; CU, Cuba; D, Germany; DK, Denmark; ES, Spain; IS, Island; PL, Poland; USA.

A phylogenetic tree resulting from an alignment of concatenated sequences, present in all analysed strains and after elimination of existing gaps, is shown in Figure 4. It revealed a very high level of genomic similarity of unrelated ST40 strains, despite their diverse origins and the time interval from <1960 to 2009. Of note, strains of a similar origin were not arranged in the same clusters. When we focused on the ST40 isolates, the core-genome based phylogenetic tree also showed an exceptional position of D32 in relation to the other sequenced ST40 isolates and furthermore its close relationship with the other Danish porcine isolate UW7742. As expected, the completely closed and publicly available *E. faecalis* genomes branch separately, supporting their assignment to different sequence types and clonal complexes based on MLST. In order to confirm the relationship between the 15 *E. faecalis* ST40 isolates with respect to their core genome, additional phylogenetic analyses were performed by mapping Solexa single reads of 14 isolates against the *E. faecalis* D32 reference sequence using a mapping pipeline based on bwa. As enterococci frequently undergo chromosomal rearrangements, we first excluded SNPs which were owed to recent recombination events. This yielded a total of 1481 variable positions (SNPs), which in turn served as the basis for tree reconstruction by the PhyML algorithm. The generated tree in Additional file 5: Figure S3 revealed a highly similar structure to the previous one (Figure 4) despite the different input data supporting the reliability of both approaches. Both trees revealed exactly identical subclusters of related strains. In Additional file 5: Figure S3 the separate clustering of the two pig commensal strains D1 and D32 from Denmark is highly visible and supported by a high bootstrap value (please recall that this separation is only based on the core genome and independent of the presence or absence of MGE). The separation of the two pig commensal isolates D1 and D32 based on core genome data disproves the hypothesis of highly related pig and human endocarditis isolates as derived from PFGE analysis (Figure 1 and [64]).

#### Analyses of mobile genetic elements

All isolates featured a modularly structured and differently composed PAI, flanked by phage-related integration and excision genes (data confirmed by long template PCR according to [45]; Additional file 1: Table S1). Of note, genome mapping also revealed that the probiotic Symbioflor 1 strain contains parts of the PAI present in D32 (see Figure 3). However, in this context it is must be noted that the reference isolate D32 itself lacks substantial parts of the PAI (as compared with MMH594) including a number of virulence determinants such as *asc*-10, the cytolysin operon and the *esp* gene. Presence of parts of the PAI has been confirmed by long template PCRs performed in advance of genome sequencing experiments. It revealed a number of well-known virulence genes in many ST40 isolates (see above and Additional file 1: Table S1).

Genome analyses of the porcine strain D32 revealed a previously novel and uncharacterised genomic island with a size of 138 kb (location: 1901082 – 2036659, EFD32_1828 – EFD32_1978) integrated at the attachment site of the conjugative *vanB* transposon in V583 (EF_2282 – EF_2334) [41,42] and the m-inositol (*iol*) operon in OG1RF, respectively (see Figure 3) [55]. Genome analysis of the probiotic strain Symbioflor indicated that neither the *iol* operon of the OG1RF nor the *vanB* transposon of V583 nor the uncharacterised genomic island of the D32 strain was present [4,65,66]. Further results of bidirectional BLAST analysis and genome mapping data (Figure 3) revealed that the novel genomic island of D32 was only verifiable in UW7729, an isolate originating from fish. We used SwissProt and BLASTP analyses to identify similarities to capsule-like genes encoded by genes of the novel genomic island in D32, with similarities to genes and gene clusters described for *Streptococcus pneumonia*e and *Bacillus subtilis* (Additional file 2: Table S4).

*Prophages.* We compared phage content by mapping against the D32 reference genome (phiFL4A, phiFL1A, B025, phiEF11 [incomplete]). Strain-specific phage patterns were recognised for some of the ST40 isolates, suggesting that prophage content varied independently of the strain background (Figure 3). For example, D32 prophage 1, showing high similarity to the enterococcal phage phiFL4A (NC_013644), was also present in UW1833, isolated from human urinary tract infection, and in the human colonising strain UW6727. In relation to D32, strains isolated from blood culture and bovine mastitis differed in prophage content, whereas the other ST40 isolates showed a comparably homogeneous level of phage content (in relation to D32).

#### Plasmid content and classification

In the context of the 15 de novo sequenced strains, investigation of plasmid content indicated a certain level of diversity independently of the strain background. Plasmids could be classified by the replication initiating gene *repA* of the corresponding replicase (*rep*) families: presence of *repA* of the well-described *E. faecalis* plasmids pAD1, pCF10 and pRE25 was confirmed by Southern hybridisation (not shown). Additionally, sequencing was used to review these results and to differentiate between the amplified conserved *repA* alleles of the closely related pAD1 and pCF10. The most dominant *rep* families among the sequenced *E. faecalis* ST40 were *rep2* (pRE25-like) and *rep9* (pCF10-like), found in three (20%) and six isolates (40%), respectively.

#### CRISPR/cas

Regarding the two CRISPR loci identified in OG1RF, our sequence and PCR results indicated that all of the selected ST40 genomes possessed the CRISPR1-*cas* and CRISPR2 loci, the latter lacking the functional *cas* genes. An exception is represented by the genome of UW7729, where only the CRISPR2 locus was present (Additional file 2: Table S5). Our detailed analyses of CRISPR2 loci showed that all strains of the sequenced ST40 subgroup possessed three identical spacers, whereby two of those were also present in OG1RF. In D32 where the CRISPR-*cas* sequences were completely available, several spacers showed homology with enterococcal phages such as phiEf11, phiFL3A and SAP6, being different from prophages found in the genome sequence of D32 (see above). The exception was phiEf11 which was present in the genome of D32; however, the phage sequence was incomplete (data not shown in details). One spacer in D32 was identical to a hypothetical protein pLG2-0017 of *E. faecalis* plasmid pLG2 (gb|HQ426665.1|)(*rep1* plasmid family; data not shown), which is not in conflict with the plasmid in D32 which is of *rep2* plasmid type.

### Utilisation of carbon sources

We postulated that the different origins and habitats of the isolates might be recovered by minor, host-specific differences in their metabolic properties as described for isolates of *E. faecium* [67]. To determine supposed differences in their metabolic profiles, Biolog MicroArray™ analyses were performed. For reasons of simplification, data values in Additional file 2: Table S6a and b were replaced by colour codes. Utilisation of various carbon sources under aerobic conditions did not show significant differences between the 15 sequenced *E. faecalis* ST40 isolates. No obvious association between origin or host and utilisation of different carbon sources was detected.

In general, results were in line with carbon utilisation patterns used for species and genus identification or as described previously [68,69]. All strains were capable of growing on trehalose, N-acetyl-glucosamine, glycerol, mannitol, glucose, lactose, sucrose and fructose-6-phosphate. They were all capable of fermenting ribose as a C5 sugar, malate (as an intermediate of the citric acid cycle) and dihydroxyaceton (as an intermediate product of fructose metabolism). No strains grew on melibiose, arabitol and methyl-D-glucoside, as expected. We only noticed a few discrepancies; for instance, according to Devriese and colleagues [69] *E. faecalis* should not ferment L-arabinose, whereas all ST40 *E. faecalis* as well as the reference strains V583 and OG1RF did. Utilisation of D-xylose is given as ‘mainly negative’, but all tested strains including our reference strains were positive. Reference isolate V583 utilised cellobiose, fructose, lactose, glucose, galactose, glycerol, maltose, mannitol, mannose, ribose, sucrose and trehalose as described [42]. One of the minor differences noticed was variable utilisation of myo-inositol (m-inositol). Belonging to one of nine isomers of the inositol group, m-inositol is used as a sole carbon source by many soil and plant microorganisms through degradation into glyceraldehyde-3-phosphate [65]. As already described, the novel and uncharacterised genomic island (138 kb) of D32 was integrated at the attachment site of the m-inositol operon in OG1RF, which consists of 10 genes. Biolog MicroArray™ analyses showed that OG1RF, but not V583 and D32, was able to utilise m-inositol. For all the other sequenced ST40 strains, the presence (but not the genomic localisation) of the *iol* operon (covering genes encoding enzymes for inositol utilisation) could be confirmed by PCR (data not shown) which was in line with a positive result of m-inositol utilisation in the Biolog MicroArray™ assay (Additional file 2: Table S6a).

### Comparative assessment of the pathogenic potential of closely related *E. faecalis* isolates

Molecular and phenotypic pre-characterisations revealed a high level of similarity between the porcine, commensal D32 and a human clinical endocarditis isolate UW7709, both from Denmark. Ability to adhere to human epithelial cells and to cause pathogenic effects in selected animal models was analysed for these two related ST40 strains.

#### In vitro growth kinetics

Over the course of 24 hours, optical density and the corresponding bacterial counts were determined (not shown). Measurement of the optical density suggested that D32 grew faster than UW7709; however, D32 also showed a tendency to clump in liquid culture. Thus, we also determined the bacterial counts, finding a comparatively similar growth rate of *E. faecalis* strains D32 and UW7709.

#### In vitro biofilm formation

**Figure 5:**
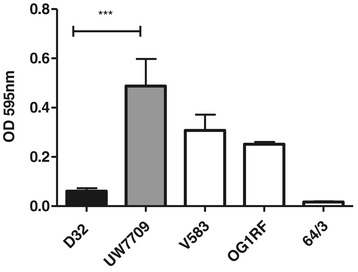
***In vitro*** **biofilm formation.** Biofilm formation of *E. faecalis* strains D32, UW7709 and the internal controls V583, OG1RF and *E. faecium* strain 64/3 on a synthetic surface was investigated by using polystyrene plates. After incubation in TSB for 24 hours, produced biofilms of adherent bacteria were stained with crystal violet. Bars represent the mean values of six or three (D32) replicates ± SEM. *** significant *P*-value < 0.0005, unpaired two-tailed t-test.

*E. faecalis* strain UW7709 showed a significantly enhanced biofilm production compared with strain D32 (Figure 5). Results of biofilm formation were also compared with the corresponding genotype. Genomes of both strains harboured *fsrB* and *gelE* genes in combination with expression of an active metalloprotease GelE. No correlation between the presence of *esp* or other biofilm-enhancing factors, such as the *ebpABC* or *epa* locus, and the in vitro capacity of biofilm formation was detected. The *esp* gene was absent in both genomes, whereas both genomes harboured the *ebp* and *epa* locus.

#### Adherence to Caco-2 cells

**Figure 6:**
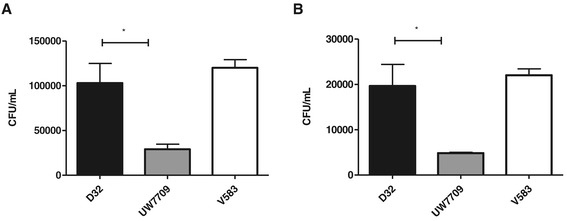
**Adhesion ability to Caco-2 cells.** To analyze the potential of adhesion, a monolayer of Caco-2 cells was incubated with a bacterial cell to epithelial cell ratio of **(A)** 1000:1 and **(B)** 100:1 for two hours with the respective strain. Data represent the mean values ± SEM. * significant *P*-value < 0.05, unpaired two-tailed t-test.

A monolayer of colonic epithelial cells (Caco-2) was incubated with *E. faecalis* strains D32 and UW7709 to test adhesion to human intestinal cells in vitro. In summary, adherence of D32 to Caco-2 cells was approximately three to four times higher than adhesion of UW7709 and similar to V583 (Figure 6).

#### Galleria mellonella model

**Figure 7:**
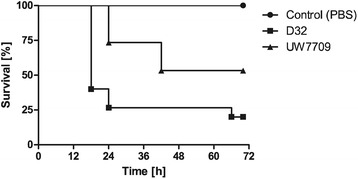
**Pathogenicity of** ***E. faecalis*** **D32 and UW7709 in a** ***Galleria mellonella*** **model.** Death rates of *G. mellonella* larvae after injection with *E. faecalis* strains D32 (real infectious dose: 1.7 × 10^5^ CFU per larvae) and UW7709 (real infectious dose: 2.8 × 10^5^ CFU per larvae), respectively. PBS injection served as a negative control. One representative experiment of three independent experiments is shown. Data are displayed by as Kaplan-Meier plot survival curves. Statistical significance (p < 0.05) was determined by the Log-rank (Mantel-Cox) test.

The insect larva *G. mellonella* is an alternative model for the study of bacteria-host interactions, and shows a complex immune reaction consisting of both cellular and humoral responses. Analysis of pathogenicity of isolates D32 and UW7709 in this model showed that D32 was more rapidly lethal for *G. mellonella* and pathogenicity of D32 was generally increased in comparison with UW7709 (Figure 7).

#### Murine bacteraemia model

**Figure 8:**
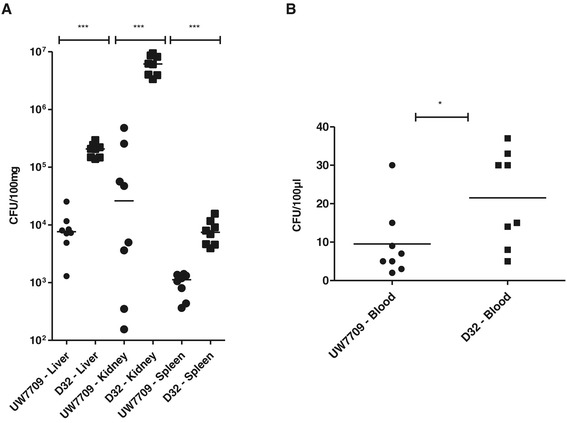
**Growth rates of** ***E. faecalis*** **UW7709 and D32 in a mouse bacteremia model.** Eight female BALB/c mice were infected via the tail vein with *E. faecalis* strains UW7709 or D32 (5 × 10^8^ CFU). After 48 hours, mice were sacrificed and bacterial counts in **(A)** liver, kidneys and spleen, as well as, in **(B)** blood were determined. Data represent the individual bacterial burdens and the geometric mean values. Asterisks indicate significant *P* values calculated by using Mann–Whitney test (*P < 0.05, ***P < 0.0005).

With the bacteraemia mouse model, D32 showed significantly enhanced bacterial recovery rates from liver, kidneys, spleen and blood in comparison with UW7709 (Figure 8).

## Discussion

### Molecular, genomic and phenotypic strain characterisation reveals a high level of similarity among diverse *E. faecalis* ST40 isolates

*Sma*I macrorestriction patterns in combination with analyses of the presence and expression of the described virulence-associated genes by PCR revealed a high level of similarity among the rather diverse collection of ST40 isolates (Additional file 1: Table S1). This suggested that strains of this clonal group did not show specific genomic characteristics or differences according to their host, context (commensal/clinical), time, and geography. The finding that major differences in content mainly derive from a diverse acquisition and composition of the various MGE still holds after whole genome sequencing of a subset of 15 isolates from this collection (Figure 4). This indicates that genomic variability within clonal types or lineages may be limited, which is in line with previous reports [49] and which does not contradict the general observation of a high level of recombination at the level of *Enterococcus faecalis* species. When we performed an SNP-based phylogenetic analysis of the 15 whole genomes an accumulation of SNPs in distinct genomic regions (mainly non-coding regions) pointed to a certain amount of recombination that most probably accompanied the exchange of MGEs. However, the exclusion of recombinational SNPs did not result in a different phylogeny (Figure 4 and Additional file 5: Figure S3), again suggesting that recombination within clonal lineage ST40 is minor.

It has been suggested that ‘ecotypes’ defined by specific mobile elements may be identified within high-risk lineages or in lineages with variable CRISPR-*cas* status such as ST40 and ST21 [41]. Environmental isolates exemplified acquisition of distinct plasmids conferring additional metabolic features such as raffinose fermentation in *E. faecium* [67]. We did not find any support in a generation of ecotypes or an adaptation to the corresponding origin or host by distinct metabolic features based on genome content which were confirmed by BIOLOG analyses (Additional file 2: Table S6a/b). Nevertheless, utilisation of sugars and other carbon sources was only slightly variable and if so, only variable for individual strains independent of their origin. Differences in carbon utilization patterns could partly be linked to genome content such as for utilising m-inositol (see below). Previous studies concluded that xylose fermentation is limited among *E. faecalis* in general, since, for instance, none of the 10 reference isolates was capable of utilising it [70]. In our case, all sequenced *E. faecalis* isolates were capable of growing on xylose, suggesting a lineage-specific property of ST40 isolates. Recent systemic studies described a model where, in the state of susceptibility to infection by *Clostridium difficile* caused by high antibiotic selective pressure, sugar alcohols are enriched in the gut metabolome [71]. Bacteria capable of utilising these sugar alcohols may have a definite growth advantage under these specific conditions which is known for *E. faecalis* and which was dedicated until now to favourable natural and acquired resistances to the used antibiotics. In particular, sugar alcohols such as mannitol and sorbitol increased several hundred-fold under the test conditions and utilisation of these sugars was also strongly positive in all our tested strains (lactitol was only positive among ST40 isolates; Additional file 2: Table 6a/b). However, this does not seem to be a general property, since although all tested *E. faecalis* strains were capable of utilising arabinose they were incapable of utilising arabitol. It should be emphasised that BIOLOG analyses in principle detect utilisation of energy sources via respiration and reduction of the tetrazolium dye and thus do not directly detect growth of microorganisms.

In a previous paper we (G.W., M.Z.) investigated group D streptococci in cases of bovine mastitis in German dairy cows [10]. Strains of ST40 predominated among *E. faecalis*, emphasising its prevalence as a common and frequent strain type also among dairy cows capable of causing mild and acute clinical cases of bovine mastitis.

Our data showed that previously described virulence-associated genes, including *fsrABDC*-regulated *gelE*, *cylM*, and *esp*, as well as clinically relevant antibiotic resistance traits, are also present in ST40 isolates from non-clinical sources. Formation of biofilm (in vitro) could not be correlated with the presence of described and putative biofilm-associated genes such as *esp* and *asc-10*. As mentioned by McBride *et al*. [39] all isolates of this most common sequence type ST40 were non-encapsulated and were also characterised as cps type 1.

Differences between the presence and expression of single markers were especially described for the *E. faecalis* cytolysin and the gelatinase in previous papers [14,15,72,73]. We did not notice any discrepancy between the presence of a gene and its expression in our collection of ST40 *E. faecalis* strains which was always consistent (Table 4).

### Detailed genomic comparisons of 15 ST40 *E. faecalis* strains identified a modularly structured PAI and a novel genomic island in isolate D32

On the basis of our previous characterisations, a subset of 15 strains, representing the diversity of the collection, was chosen for de novo pyrosequencing by standard Roche 454 GS-FLX technology. Because no complete ST40 genome sequence was publicly available, we decided to resolve the complete genome sequence of a single pig commensal strain D32 for use as a ST40 template for detailed genome comparisons. After scaffolding with an 8 kb long-paired end (LPE) library, the remaining gaps and assembly ambiguities (‘InDel’ errors) were corrected by Sanger sequencing. We gained experience in the use of the two assembly software tools Newbler and Celera, and generated two and one chromosomal scaffolds, respectively. Usage of Celera resulted in fewer misassembled regions, whereas the assembly of repetitive sequences was especially difficult for Newbler. We generated quite positive experience by using PacBio technology for de novo genome sequencing and assemblies and would favour this approach again for upcoming projects. In brief, we were able to generate a single chromosomal scaffold of 2.7 MB by using only the PacBio approach (long read and short read libraries) of an *E. faecium* isolate and by using the HGAP algorithm (assemblies done by Celera and polishing by Quiver software; https://github.com/PacificBiosciences/Bioinformatics-Training/wiki/Large-Genome-Assembly-with-PacBio-Long-Reads; project in cooperation with R. Vogelsang, G. Kuhn and F. Boellmann from Pacific Biosciences, previously unpublished data; last access 20.07.2014).

In agreement with McBride *et al*. [39], we also demonstrated that all of the isolates featured a modularly structured pathogenicity island, varying independently of the strain background and flanked by putative phage-related integrase and excisionase genes [14]. The D32 PAI contained a bile acid hydrolase (*cbh*) and lactose metabolic pathway genes (*lac*ABCDEFG). It lacked the common markers present in the original prototype PAI of MMH594 which were mainly associated with virulence, like *esp,* the *asc-10* gene and the cytolysin operon. In the case of D32, composition of the PAI may suggest a putative adaptation to pig’s intestine by the presence of bile acid hydrolase (*cbh*) and the complete lactose metabolic pathway genes (*lacABCDEFG*). Detailed analyses of the genetic components of the PAIs of the 14 *E. faecalis* ST40 draft genomes were limited by the existence of intra-chromosomal gaps. However, the presence and absence of PAI components/clusters were available from results of long PCR analyses, which showed variability independent from the host (Additional file 1: Table S1). Variability is derived from partial or complete mobility of the PAI, as demonstrated recently [45,46,74]. These results again emphasise that the PAI evolves through HGT and recombination in a much faster way than the relatively conserved core genome [32,41].

A so far unknown genomic island was identified as integrated within the genome of the porcine commensal strain D32. Apart from several genes with unknown identity, it contains a genomic cluster probably associated with exopolysaccharide synthesis. Consisting of different sugars, the extracellular polysaccharides (EPS) are polymeric complex structures covalently bound to the cell surface or released in its environment [75,76]. Importantly, conserved *E. faecalis* cell wall polysaccharides are the rhamnopolysaccharide Epa, encoded by the enterococcal polysaccharide biosynthesis locus *epa*, and the capsular polysaccharide Cps (*cps* cluster), consisting of galactose, glucose and phosphate [77]. All ST40 strains were non-encapsulated and are grouped as CPS type 1. Although the presence of *cps* genes does not necessarily result in the expression of the corresponding capsule phenotype [78], genes related to cell surface structures are enriched with strains of CC2 [35,39,79].

Investigations regarding the integration site of the novel GI showed that it is integrated within a conserved *attL*/*attR* attachment site, previously described as a ‘hot spot’ for rearrangements or new integrations. It is suggested that two different integration events resulted in the presence of an *iol* operon in OG1RF and a conjugative *vanB* transposon in V583 [42,65]. The attachment region is missing in the Symbioflor 1 strain [4]. Our sequencing results demonstrated that all of the ST40 strains, with the exception of D32, harbour an *iol* operon comparable to OG1RF, coupled with identical phenotypes of m-inositol utilisation (see Additional file 2: Table S6a/b). M-inositol is a substance abundant in nature and its utilisation was suggested recently as an auxiliary trait promoting fitness in lactic acid bacteria such as lactobacilli [80]. Genome data in combination with results of Biolog MicroArray™ analyses of the strain UW7729, isolated from fish, suggested that the *iol* operon was co-integrated next to the novel GI. However, the presence of intra-chromosomal gaps within this genomic region limited our opportunities to display the co-localisation in more molecular detail.

### Resolving the plasmid and phage content and prevalence of genomic islands requires techniques in addition to illumina sequencing

Suitable software tools which filter for plasmid sequences, generated by 454 or the Illumina’s Solexa sequencing technology, are limited, as are approaches to identifying entire plasmids from de novo assembled genomic DNA. Thus, ‘classical’ molecular methods, such as plasmid isolation and analysis of the plasmid size by S1 nuclease PFGE in combination with PCR- and hybridisation-based *rep*-typing, were used as more meaningful and informative approaches. In combination with genomic mapping of the ST40 draft genomes against the D32 template, results revealed a comparably high level of diversity in plasmid content [81] and strain-specific phage patterns (Figure 3). Integration of bacteriophages might have a significant impact on niche adaptation. Duerkop *et al*. [82] hypothesised that bacteriophages, especially a composition of different bacteriophages, cause bacterial dominance within the microbial ecosystem, where competition for nutrients especially plays a crucial role. Integration of the bacteriophage into a promoter region might result in an increase of expression of putative virulence-associated genes [83]. Surface-exposed wall teichoic acids might be used as specific identification markers for bacteriophages, enabling HGT of virulence and resistance genes across genus-specific barriers of Gram-positive pathogens [84].

The rep-typing scheme for determining plasmid types demonstrated that *rep*-types 2 and 9 were predominant in the ST40 strain collection but disclosed also the presence of some new plasmids [55,85,86]. These results indicated that recombination and rearrangements of chromosomal and other plasmid DNA might contribute to mosaic-related structures, whereby an adaptation to changing environmental conditions is mediated [87].

A subset of ST40 strains has been analysed at a partner’s laboratory by microarray hybridisation analyses of isolates of *E. faecalis* and *E. faecium* dedicated to detecting specifically mobile genetic elements and resistance genes for another study [88]. Only isolates of ST40 harboured possible functional CRISPR-*cas* systems which were not detected, for instance, in hospital*-*associated *E. faecalis* ST6 isolates (CC2). Whereas resistance determinants were evenly distributed among different strains and ST types, isolates of ST40 in particular showed an accumulation of antibiotic, metal and biocide resistance determinants. A link to the presence of corresponding mobile elements such as transposons, conjugative transposons and plasmids frequently carrying these resistance genes was noticeable, suggesting that the presence of CRISPR-*cas* is highly specific and does not generally exclude accumulation of resistance determinants or mobile genetic elements as suggested recently [89].

### Using different in vivo models to analyse adhesion and pathogenicity revealed a higher pathogenic potential of D32 in comparison with UW7709

Increased adherence to biotic surfaces such as that of D32 to Caco-2 cells (Figure 6) does not necessarily correlate with an enhanced capacity of biofilm formation in vitro (regarding abiotic surfaces; Additional file 3: Figure S1), a fact that has been addressed already in previous papers [90,91]. So far described regulators, such as Fsr [92], EbpR [93], EbrA [94], and PerA [95], are suggested to influence biofilm formation and, as demonstrated recently, other genes of the conserved core genome could also be involved in biofilm expression and gene regulation [94].

A number of different in vivo models were utilised to compare the in vivo survival and pathogenic potential of the two related *E. faecalis* strains D32 and UW7709. Growth in vitro did not differ between these two strains. In the insect larvae *Galleria* model, D32 was more rapidly lethal and pathogenicity of D32 was generally higher compared with UW7709 (Figure 7). Similar results were generated when recovery rates of bacterial counts were compared for the murine bacteraemia model with significantly enhanced amounts of D32 in comparison with UW7709 isolated from liver, kidneys, spleen and blood (Figure 8). Results were confirmed with a rat endocarditis model in a partner’s lab (T.S and J. H.) (not shown) [96]. During the course of this study, we tested two other avian models of infection at a partner’s laboratory (H.M.H, R.H.), which also gave similar results (not shown) [97]. Taken together, all used animal models of infection and pathogenicity proclaimed a higher pathogenic potential of the pig isolate D32 in comparison with the human endocarditis isolate UW7709. Obvious differences in the genome content of both isolates mainly included the presence or absence of a few prophage sequences and the novel genomic island in D32 potentially encoding a cluster of polysaccharide capsule genes (Figure 3). Arguing that this novel genomic island increases pathogenicity of D32 in the tested animal models will require targeted knockout experiments by deleting this novel 134 kb island in the strain background of D32 and subsequent analysis of the behaviour of isogenic strain pairs in the different models; such experiments are planned.

Preliminary results of secretome analyses suggested differences in the extracellular proteome of the two related strains D32 and UW7709 [98]. Differences in the secretome and the above-mentioned putative novel exopolysaccharide cluster could explain why D32 in comparison with UW7709 showed significantly different behaviour in the presented bacteria-host models [99,100].

## Conclusion

Our detailed molecular and phenotypic analyses of 42 *E. faecalis* strains of MLST type ST40 and the genomic analyses of a subset of 15 isolates revealed a minor level of genomic diversity. Isolates are highly related regarding the core genome and only demonstrated variable accessory genome content in respect of the presence and composition of the *E. faecalis* PAI, plasmids and phages, independently of their corresponding animal or human background or the context (colonisation/infection). We were unable to find any indication of niche adaptation associated with the supposed origin or clinical context of distinct ST40 strains. The animal isolate D32, whose genome was completely resolved during this study, contained a novel genomic island of 138 kb, putatively involved in exopolysaccharide synthesis (capsule formation, perhaps?). D32 showed enhanced pathogenic potential in various animal models compared with the related human endocarditis isolate UW7709. Our hypothesis suggests that different behaviour of D32 and UW7709 is associated with the presence of this novel genomic island, a supposition which needs to be proven in future analyses.

### Animal experiments

The animal welfare committees of the university of Freiburg (Regierungspräsidium Freiburg Az 35/9185.81/G-07/15) approved all animal experiments (sepsis model G-07/15; rat endocarditis model G-07/72; urinary tract infection model G-11/118).

### Availability of supporting data

All supporting information is deposited as Supplementary Figures or Tables.

## Endnote

^a^http://www.ecdc.europa.eu/en/publications/surveillance_reports/annual_epidemiological_report/Pages/epi_index.aspx

## Additional files

Additional file 1: Table S1. Main characteristics of the analysed 42 ST40 *E. faecalis* isolates. Additional file 2: Table S2. Primers used for the amplification of antibiotic resistance genes, virulence genes, parts of the *E. faecalis* PAI, plasmid replicase genes and other genes. **Table S3.** Quality report of 454 sequencing data assembled with Newbler software. **Table S4.** SwissProt and BLASTP analyses of a putative capsule-encoding region within the *E. faecalis* D32 genomic island. **Table S5.** Identification of CRISPR loci in selected *E. faecalis* ST40 strains by PCR. **Table S6.** Aerobic utilization of carbon sources of Biolog MicroArray™ PM01 and PM02. Additional file 3: Figure S1. Capability of *E. faecalis* isolates of ST40 to form biofilm in vitro on polystyrene microtiter plates (Greiner Bio-one, Germany and Corning Inc., NY, USA). Isolates from UTI are marked with dark blue as well as isolates from endocarditis (blue). Strains *E. faecalis* V583, OG1RF and OG1RFK12 (OG1RF including the PAI and plasmid pLG2 [45] were used as reference isolates as well as *E. faecium* 64/3 [54] as a negative control (all marked with black). Isolate UW7742 (= D32) is the completely sequenced ST40 reference isolate showing a lower biofilm forming capability. Additional file 4: Figure S2. S1 nuclease analysis resolving plasmid content of 18 ST40 *E. faecalis* isolates. This representative gel of S1 nuclease PFGE showed the presence of none, one or two linearized plasmids. A red arrow points at a single plasmid band of the sequenced *E. faecalis* strain D32 (size of circa 75 kb). The upper bands (migrating above the 674 kb band of NCTC8325), visible in all lanes, correspond to undigested chromosomal DNA. NCTC8325 applies to *Sma*I-digested genomic DNA of *S. aureus* NCTC8325 used as a size marker in PFGE analysis. Additional file 5: Figure S3. Phylogenetic analysis of 15 *E. faecalis* ST40 genomes resulting from mapping illumina reads against the D32 reference genome. Solexa single reads were mapped onto the reference sequence of *E. faecalis* D32 by utilizing a mapping pipeline based on bwa (Steglich et al., previously unpublished [see Materials]). Altogether 10,4 % of ambiguous sites were detected compared to the reference genome and excluded from further analysis as well as SNPs resulting from recombination, thereby producing 1481 SNPs for phylogenetic calculations by the PhyML algorithm (seaview program) with a bootstrap of 1000. Metadata are given as follows: Strain no., year of isolation, origin, country: AC, animal colonizer; AI, animal infection; HC, human colonizer; HI, human infection; CU, Cuba; D, Germany; DK, Denmark; ES, Spain; IS, Island; PL, Poland; USA.
