# Supplementary material for: Comprehensive molecular, genomic and phenotypic analysis of a major clone of Enterococcus faecalis MLST ST40
Source: BMC Genomics. 2015 Mar 12;16(1):175. doi: 10.1186/s12864-015-1367-x (PMC4374294; doi:10.1186/s12864-015-1367-x)
Supplement: Additional file 2: Table S2. — Primers used for the amplification of antibiotic resistance genes, virulence genes, parts of the E. faecalis PAI, plasmid replicase genes and other genes. Table S3. Quality report of 454 sequencing data assembled with Newbler software. Table S4. SwissProt and BLASTP analyses of a putative capsule-encoding region within the E. faecalis D32 genomic island. Table S5. Identification of CRISPR loci in selected E. faecalis ST40 strains by PCR. Table S6. Aerobic utilization of carbon sources of Biolog MicroArray™ PM01 and PM02. [file 12864_2015_1367_MOESM2_ESM.docx]

**Supplementary Tables**

**Suppl. Table S2: Primers used for the amplification of antibiotic resistance genes, virulence genes, parts of the *E. faecalis* PAI, plasmid replicase genes and other genes.**

| ***Gene*** | ***GenBank***  ***Acc. No.*** | ***Primer name*** | ***Primer sequence (5' → 3')*** | ***Amplicon [bp]*** | ***Reference*** |
| --- | --- | --- | --- | --- | --- |
| *vanA* | AF516335 | vanA I-1 | TCTGCAATAGAGATAGCCGC | 377 | [[1](#_ENREF_1)] |
|  |  | vanA II-2 | GGAGTAGCTATCCCAGCATT |  |  |
| *erm*(B) | AF516335 | ermB-F | AGCCATGCGTCTGACATCTAT | 341 | [[1](#_ENREF_1)] |
|  |  | ermB-R | TGCTCATAAGTAACGGTACT |  |  |
| *tet*(M) | n.d. | tetM-F | GGTGAACATCATAGACACGC | 401 | This study |
|  |  | tetM-R | CTTGTTCGAGTTCCAATGC |  |  |
| *aacA-aphD* | n.d. | gen-F | TAATCCAAGAGCAATAAGGGC | 227 | This study |
|  |  | gen-R | GCCACACTATCATAACCACTA |  |  |
| *aadE* | AF516335 | aadE-1 | GCAGAACAGGATGAACGTATTCG | 369 | [[1](#_ENREF_1)] |
|  |  | aadE-2 | ATCAGTCGGAACTATGTCCC |  |  |
| *asc10* | EF0005 | PAIasc10-1 | GCCAAAGTGGAACGTTAAATG | 345 | [[2](#_ENREF_2)] |
|  |  | PAIasc10-2 | TCAATCCAGAAGGTCCTGTG |  |  |
| *cylM* | EF0046 | cylM-TQ1 | GATGCGTATTACTGTTGTTAGAATGAGAT | 150 | [[2](#_ENREF_2)] |
|  |  | cylM-TQ2 | GAGTCTCCCTGTGATTCTGATATAGAGTT |  |  |
| *esp* | EF0056 | esp-TIM1 | CTTTGATTCTTGGTTGTCGGATAC | 475 | [[3](#_ENREF_3)] |
|  |  | esp-TIM2 | TCCAACTACCACGGTTTGTTTATC |  |  |
| *xyl kinase* | EF0083 | PAIefc-83F | GGAGCTGATAATGCTTGTGC | 202 | [[4](#_ENREF_4)] |
|  |  | PAIefc-83R | AAGAATTACCTGCTGCCAAC |  |  |
| *gls24-like* | EF0117 | gls24-F | TGAAGCAAATTCTCCAGTAGC | 262 | [[2](#_ENREF_2)] |
|  |  | gls24-R | TGGAGTGGATGTTGAAGTAGG |  |  |
|  | AE016830.1 | PAI164 | ATGCCATGTTCAGCGAAGTTGCCAATTATC | * | [[4](#_ENREF_4),[5](#_ENREF_5)] |
|  |  | PAI167 | ATGTTGGTTGAAAGTTGCTTTTTGGCAAAC |  |  |
| *gelE* | M37185.1 | gelE-F | TATGACAATGCTTTTTGGGAT | 213 | [[3](#_ENREF_3)] |
|  |  | gelE-R | AGATGCACCCGAAATAATATA |  |  |
| *fsrB* | EF_1821* | fsrB-1 | GCATTGTTATCTATGTCGCCATACC | 397 | [[6](#_ENREF_6)] |
|  |  | fsrB-2 | GGCTTAGTTCCCACACCATC |  |  |
| *cpsA* | EF_0095 | cpsA-F | GTGTCTCCTGAAAAATCAGGCC | 383 | [[7](#_ENREF_7)] |
|  |  | cpsA-R | GTTAAAGTCAATGTAATGGGCTACC |  |  |
| *cpsB* | EF_0094 | cpsB-F | CTATCAAAACGATCTAAAATACCACC | 619 | [[7](#_ENREF_7)] |
|  |  | cpsB-R | GATTAACGTTATTAAGTTTTGAAGGCG |  |  |
| *cpsC* | EF_0093 | cpsC-F | CCAACGCTTTGCTTCTTGAATGAC | 300 | [[6](#_ENREF_6)] |
|  |  | cpsC-R | CCTGAATATCAATGTATTTGGGCAGTC |  |  |
| *cpsF* | EF_0090 | cpsF-F | GGCGATCTATTCTACCATCCGCGC | 324 | [[7](#_ENREF_7)] |
|  |  | cpsF-R | CCAAAGAAAGATATTTTGGATTGAG |  |  |
| *CRISPR1-cas* | AE016830.1 | CRISPR1-cas-F | GCGATGTTAGCTGATACAAC | 316 | [[8](#_ENREF_8)] |
|  |  | CRISPR1-cas-R | CGAATATGCCTGTGGTGAAA |  |  |
| *CRISPR1 cas_csn* | NC_017316.1 | CRISPR1-cas_csn1-F | CAGAAGACTATCAGTTGGTG | 783 | [[8](#_ENREF_8)] |
|  |  | CRISPR1-cas_csn1-R | CCTTCTAAATCTTCTTCATAG |  |  |
| *CRISPR2* | AE016830.1 | CRISPR2-F | CTGGCTCGCTGTTACAGCT | variabel | [[8](#_ENREF_8)] |
|  |  | CRISPR2-R | GCCAATGTTACAATATCAAACA |  |  |
| *CRISPR3-cas* | AE016830.1 | CRISPR3-cas-F | GATCACTAGGTTCAGTTATTTC | 225 | [[8](#_ENREF_8)] |
|  |  | CRISPR3-cas-R | CATCGATTCATTATTCCTCCAA |  |  |
| *iolB* | NC_017316.1 | iolB-F | CCATCTGGCACGCCGACAGGA | 363 | This study |
|  |  | iolB-R | GCCAGTGCACGTGATTACCGCTG |  |  |
| *iolG2* | NC_017316.1 | iolG2-F | GCGTTTGCCAGTCGGGCGAAA | 465 | This study |
|  |  | iolG2-R | TGGTACAGGTGGGCTTCATGCGT |  |  |
| *iolE* | NC_017316.1 | iolE-F | ACGGATTGGCTTTGGCCGGATCT | 363 | This study |
|  |  | iolE-R | TGGGGACAGGAGTCCAAACGACTG |  |  |
| *iolR* | NC_017316.1 | iolR-F | TCCCTAATCGCCACACTA | 379 | [[9](#_ENREF_9)] |
| PAI 1 | AF454824.1*:* C:427 | PAI164 | ATGCCATGTTCAGCGAAGTTGCCAATTATC | 1548 | [[4](#_ENREF_4)] |
|  |  | 1 PAI R | GGAAGATGGACGGTTGATGAAGCCTCAATATG |  |  |
| PAI 2a | AF454824: 240:9913 | 2a PAI F | CAGTTGTCGAATACGATGCATGTCCCAGCC | 9674 | [[4](#_ENREF_4)] |
|  |  | 2a PAI R | AAACCAAAGGAACCGAAACGGAAAAACTTAGCATGG |  |  |
| PAI 2b | AF454824: 9869:15934 | 2b PAI F | TTTAACCAGCCATGCTAAGTTTTTCCGTTTCGGTTC | 6066 | [[4](#_ENREF_4)] |
|  |  | 2b PAI R | TTTGAAATAATCTCCAACTTTTCCCCCGTTCCACAC |  |  |
| PAI 2c | AF454824: 14033:21999 | 2c PAI F | AACCATAAAAAGGAACGGAGGGAGCACAACAAAAGG | 7697 | [[4](#_ENREF_4)] |
|  |  | 2c PAI R | ACTTGCAGTGTGACTGTCTGTCGTAACTTCACC |  |  |
| PAI 3a | AF454824: 21566:32079 | 3a PAI F | CTCGTCCGTAACGATCTGTTTTATCGCCCTTATC | 11645 | [[4](#_ENREF_4)] |
|  |  | 3a PAI R | TCAAGTCCGTACAACAGGCACTTTCTTTATCAAGC |  |  |
| PAI 3b | AF454824: 31320:42730 | 3b PAI F | GAAGGCCGTTGCCAATTTTGCATTAGCTTGC | 11411 | [[4](#_ENREF_4)] |
|  |  | 3b PAI R | TCCTAAGCCTATGGTAAAACATGCTGGAGTTGTCTC |  |  |
| PAI 4a | AF454824: 42395:53473 | 4a PAI F | CAAGGTAGTGGAGATGTTCAGGCTGAGACAACAC | 11079 | [[4](#_ENREF_4)] |
|  |  | 4a PAI R | CGGATGTTACTTCTGCTGGACTTAAAACAATCCC |  |  |
| PAI 4b | AF454824: 53440:65000 | 4b PAI F | GGGATTGTTTTAAGTCCAGCAGAAGTAACATCCG | 11561 | [[4](#_ENREF_4)] |
|  |  | 4b PAI R | ACGCCAAGCACAAGGGATAAAGATTGCGAAAG |  |  |
| PAI 5a | AF454824: 64944:75572 | 5a PAI F | GGACGACCTTTATAGACGCCGTTTGCTTTCG | 10629 | [[4](#_ENREF_4)] |
|  |  | 5a PAI R | AGTCCCCTTTTTCTGCCATGACACCAGTTAAAATC |  |  |
| PAI 5b | AF454824: 74158:85588 | 5b PAI F | GCTGTGGTCAAGATAGATGGGAAAGAGATTGAGCG | 11431 | [[4](#_ENREF_4)] |
|  |  | 5b PAI R | GGATCTGAACCGTCTTGTGTCATAGTGTGCCAG |  |  |
| PAI 6a | AF454824: 85547:94661 | 6a PAI F | TGTAGCATACTGGCACACTATGACACAAGACGG | 9115 | [[4](#_ENREF_4)] |
|  |  | 6a PAI R | CGTGCCCCTAATTACCATAGAGATAGTCGCGTTG |  |  |
| PAI 6b | AF454824: 93984:102421 | 6b PAI F | TGGTAAACGCTGCTCCTGAAATGAAGAGTTTGAC | 8432 | [[4](#_ENREF_4)] |
|  |  | 6b PAI R | AGGTTTGATACGCAACTACCTTTCCCAACTGACG |  |  |
| PAI 7a | AF454824: 101954:113046 | 7a PAI F | TTTTGGGACAGGAACGCTATCAGTTAACGATTGC | 10821 | [[4](#_ENREF_4)] |
|  |  | 7a PAI R | CCTGCGGTCAAGCACAGTTGCCTTATCTTAG |  |  |
| PAI 7b | AF454824: 113008:126865 | 7b PAI F | ATTAAAGTCAAAAGAGACTGTTACTTGTGCGCCCTG | 13858 | [[4](#_ENREF_4)] |
|  |  | 7b PAI R | TCAGCAAACTAAGATAAGGCAACTGTGCTTGACC |  |  |
| PAI 8a | AF454824: 125344:136351 | 8a PAI F | TGCTTTAGTGGGTCGTACTAACGGAACAATAG | 11008 | [[4](#_ENREF_4)] |
|  |  | 8a PAI R | CAAACAACACGTCGTCGATCTTTACCTTG |  |  |
| PAI 8b | AF454824: 135337:146384 | 8b PAI F | CACCAATGCACATAATCAAACAATTCTAGGCGTAG | 11048 | [[4](#_ENREF_4)] |
|  |  | 8b PAI R | GTGGACAAGCACAGTCACAATTAGAAGCAATG |  |  |
| PAI 9 | AF454824: 146272:C | 9 PAI F | CATCATTTCTTCAGCAAATTGGTTGGCACGC | 8298 | [[4](#_ENREF_4)] |
|  |  | PAI167R | ATGTTGGTTGAAAGTTGCTTTTTGGCAAAC |  |  |

| rep-pCF10 | AY885841 | repCF10-1 | GCTCGATCARTTTTCAGAAG | 201 | [[10](#_ENREF_10)] |
| --- | --- | --- | --- | --- | --- |
|  |  | repCF10-2 | CGCAAACATTTGTCWATTTCTT |  |  |
| rep-pRE25 | X92945 | repRE25-1 | GAGAACCATCAAGGCGAAAT | 630 | [[10](#_ENREF_10)] |
|  |  | repRE25-2 | ACCAGAATAAGCACTACGTACAATCT |  |  |

Suppl Table S3: Quality report of 454 sequencing data assembled with Newbler software.

| ***Isolate*** | ***Origin*** | ***GC content***  ***[%]*** | ***Number of***  ***contigs*** | ***Calculated genome***  ***size [bp]*** | ***Coverage***  ***[n-fold]*** |
| --- | --- | --- | --- | --- | --- |
| UW6149 | B | 37.51 | 163 | 3,239,149 | 13.92 |
| UW2860 | B | 37.51 | 104 | 3,062,478 | 15.78 |
| UW6724 | HC | 37.31 | 298 | 3,050,235 | 10.04 |
| UW7761 | B | 37.51 | 178 | 2,912,463 | 11.60 |
| UW7777 | HC | 37.55 | 133 | 2,945,792 | 34.32 |
| UW7780 | HC | 37.35 | 267 | 3,059,826 | 15.60 |
| UW7753 | B | 37.32 | 148 | 3,158,895 | 13.54 |
| UW1833 | U | 37.10 | 228 | 3,190,695 | 17.29 |
| UW7779 | HC | 37.21 | 143 | 3,024,279 | 22.45 |
| UW7729 | AC | 37.26 | 370 | 2,946,024 | 11.16 |
| UW7801 | M | 37.51 | 218 | 2,900,593 | 10.55 |
| UW6727 | HC | 37.02 | 236 | 3,275,508 | 18.39 |
| D32 | AC | 37.63 | 71 | 2,840,807 | 24.36 |
| UW7709 | E | 37.26 | 94 | 2,921,715 | 23.13 |
| UW7742 | AC | 37.34 | 124 | 2,889,018 | 22.59 |

Genomes were *de novo* sequenced and assembly was done with Newbler assembler software. Assembly of only two strains D32 and UW7709 resulted in less than 100 contigs. Genome size varied between 2.8 to 3.3Mbp, irrespective of the clinical or non-clinical background of the isolates. Coverage of the genomes was between 10 to 34-fold; AC, animal colonizer; B, blood culture; E, endocarditis; HC, human colonizer; M, bovine mastitis; U, urine.

Suppl. Table S4: SwissProt and BLASTP analyses of a putative capsule-encoding region within the *E. faecalis* D32 GI.

| ***Locus Tag***  ***EFD32_*** | ***annotated product name*** | ***SwissProt - best hits^a^*** | ***BLASTP - best hits^a^***  ***S. pneumoniae TIGR4*** | ***BLASTP - best hits^a^***  ***B. subtilis 168*** |
| --- | --- | --- | --- | --- |
| 1891 | UDP-glucose 6-dehydrogenase | UDP-glucose 6-dehydrogenase  (0.0, 64%, 100%) | None | UDP-glucose dehydrogenase  (2e-41, 29%, 86%) |
| 1892 | Glycosyl transferase, group 1 family protein | Glycosyltransferase Gtf1  (2e-07, 26%, 45%) | Group 1 glycosyl transferase  (2e-08, 26%, 43%) | UDP-glucose: polyglycerol phosphate alpha-glucosyltransferase  (1e-07, 30%, 36%) |
| 1895 | UDP-glucose 4-epimerase | UDP-glucose 4-epimerase  (2e-163, 65%, 98%) | Capsular polysaccharide biosynthesis protein Cps4J  (1e-169, 65%, 98%) | EpsC, UDP-sugar epimerase  (1e-42, 35%, 88%) |
| 1897 | Undecaprenyl-phosphate galactosephosphotransferase | Undecaprenyl phosphate N,N'-diacetylbacillosamine 1-phosphate transferase  (3e-77, 58%, 96%)^b^ | Capsular polysaccharide biosynthesis protein Cps4E  (4e-38, 47%, 91%) | Phosphotransferase  (6e-78, 56%, 96%)^c^ |
| 1901 | Putative tyrosine-protein phosphatase CapC | Tyrosine-protein phosphatase YwqE  (3e-61, 41%, 100%) | Capsular polysaccharide biosynthesis protein Cps4B  (9e-25, 27%, 88%) | YwqE, protein tyrosine-phosphatase  (3e-63, 41%, 100%) |
| 1902 | Tyrosine-protein kinase YwqD | Tyrosine-protein kinase YwqD  (2e-69, 50%, 93%) | Capsular polysaccharide biosynthesis protein Cps4D  (5e-40, 38%, 88%) | YwqD, protein tyrosine kinase  (2e-70; 50%, 93%) |
| 1903 | Capsular polysaccharide synthesis enzyme | Probable capsular polysaccharide biosynthesis protein YwqC  (8e-42, 38%, 93%) | Transcriptional regulator  (1e-70, 41%, 94%) | YwqC, modulator of YwqD protein tyrosine kinase activity  (5e-41, 38%, 93%) |
| 1904 | Transcriptional regulator lytR | Transcriptional regulator LytR  (3e-163, 72%, 96%) | Transcriptional regulator  (1e-70, 41%, 94%) | Membrane-bound transcriptional regulator LytR  (4e-81, 41%, 90%) |

^a^ Values in parentheses are E value, % identity, % query coverage; ^b^ second best hit: uncharacterized sugar transferase EpsL (7e-76, 56%, 96%); ^c^ second best hit: TuaA, putative undecaprenyl-phosphate N-acetylgalactosaminyl 1-phosphate transferase (4e-26, 40%, 69%).

Suppl. Table S5: Identification of CRISPR loci in selected *E. faecalis* ST40 strains by PCR.

| ***Isolate*** | ***cas_csn1*** | ***CRISPR1-cas*** | ***Size [bp]*** | ***CRISPR2*** | ***No. of spacer*** |
| --- | --- | --- | --- | --- | --- |
| V583 | - | - | 773 | + | 2 |
| OG1RF | + | + | 1031 | + | 7 |
| UW1833 | + | + | 870 | + | 4 |
| UW6724 | + | + | 886 | + | 4 |
| UW6727 | + | + | 857 | + | 4 |
| UW7777 | + | + | 864 | + | 4 |
| UW7779 | + | + | 892 | + | 4 |
| UW2860 | + | + | 891 | + | 4 |
| UW6149 | + | + | 892 | + | 4 |
| UW7801 | + | + | 861 | + | 4 |
| UW7729 | - | - | 927 | + | 5 |
| UW7709 | + | + | 834 | + | 4 |
| UW7742 | + | + | 988 | + | 6 |
| D32 | + | + | 1432 | + | 13 |
| UW7761 | + | + | 915 | + | 5 |
| UW7753 | + | + | 860 | + | 4 |
| UW7780 | + | + | 831 | + | 4 |

Analog to [[8](#_ENREF_8)], presence of CRISPR loci corresponding to OG1RF CRISPR1-*cas* and CRISPR2 loci [[9](#_ENREF_9),[11](#_ENREF_11)] was checked by PCR. CRISPR2 locus was also sequenced to identify the integrated spacer. Analog to [[11](#_ENREF_11)], existence of an empty CRISPR2 locus of the hospital-adapted V583 strain, also lacking the functional *cas* genes [[8](#_ENREF_8),[11](#_ENREF_11)] was demonstrated.

Suppl. Table 6a: Aerobic utilization of carbon sources of Biolog MicroArray™ PM01.

Mean area values were calculated of three independent experiments performed at 37°C for a 72h incubation time. For reasons of simplification, mean area values have been replaced by a color code. Grey color indicated no or only weak substrate utilization (mean area value below 10,000) and pink symbolized middle values (mean area value between 10,000 and 19,999). The green color represented strains with a high capability of utilization of the respective carbon source (mean area value greater or equal to 20,000). The red box highlighted m-Inositol as the carbon source of main interest.

Suppl. Table 6b: Aerobic utilization of carbon sources of Biolog MicroArray™ PM02.

Mean area values were calculated of three independent experiments performed at 37°C for a 72h incubation time. For reasons of simplification, mean area values have been replaced by a color code. Grey color indicated no or only weak substrate utilization (mean area value below 10,000) and pink symbolized middle values (mean area value between 10,000 and 19,999). The green color represented strains with a high capability of utilization of the respective carbon source (mean area value greater or equal to 20,000).

**References**

1. Werner G, Hildebrandt B, Witte W (2003) Linkage of *erm*(B) and *aadE-sat4-aphA*-3 in multiple-resistant *Enterococcus faecium* isolates of different ecological origins. MicrobDrug Resist 9 Suppl 1: S9-16.

2. Shankar N, Baghdayan AS, Gilmore MS (2002) Modulation of virulence within a pathogenicity island in vancomycin-resistant Enterococcus faecalis. Nature 417: 746-750.

3. Vankerckhoven V, Van Autgaerden T, Vael C, Lammens C, Chapelle S, et al. (2004) Development of a multiplex PCR for the detection of asa1, gelE, cylA, esp, and hyl genes in enterococci and survey for virulence determinants among European hospital isolates of Enterococcus faecium. J Clin Microbiol 42: 4473-4479.

4. Laverde Gomez JA, Hendrickx AP, Willems RJ, Top J, Sava I, et al. (2011) Intra- and interspecies genomic transfer of the Enterococcus faecalis pathogenicity island. PLoS One 6: e16720.

5. Laverde Gomez JA (2011) Horizontally Transferable Elements among Enterococci. [Dissertation (Dr. rer. nat.)]: Technischen Universität Carolo-Wilhelmina zu Braunschweig.

6. McBride SM, Fischetti VA, Leblanc DJ, Moellering RC, Jr., Gilmore MS (2007) Genetic diversity among Enterococcus faecalis. PLoS One 2: e582.

7. Hancock LE, Gilmore MS (2002) The capsular polysaccharide of Enterococcus faecalis and its relationship to other polysaccharides in the cell wall. Proc Natl Acad Sci U S A 99: 1574-1579.

8. Palmer KL, Gilmore MS (2010) Multidrug-resistant enterococci lack CRISPR-cas. MBio 1: e00227-00210.

9. Bourgogne A, Garsin DA, Qin X, Singh KV, Sillanpaa J, et al. (2008) Large scale variation in Enterococcus faecalis illustrated by the genome analysis of strain OG1RF. Genome Biol 9: R110.

10. Jensen LB, Garcia-Migura L, Valenzuela AJ, Lohr M, Hasman H, et al. (2010) A classification system for plasmids from enterococci and other Gram-positive bacteria. J Microbiol Methods 80: 25-43.

11. Horvath P, Coute-Monvoisin AC, Romero DA, Boyaval P, Fremaux C, et al. (2009) Comparative analysis of CRISPR loci in lactic acid bacteria genomes. Int J Food Microbiol 131: 62-70.
