# Supplementary material for: Comprehensive molecular, genomic and phenotypic analysis of a major clone of Enterococcus faecalis MLST ST40
Source: BMC Genomics. 2015 Mar 12;16(1):175. doi: 10.1186/s12864-015-1367-x (PMC4374294; doi:10.1186/s12864-015-1367-x)
Supplement: Additional file 3: Figure S1. — Capability of E. faecalis isolates of ST40 to form biofilm in vitro on polystyrene microtiter plates (Greiner Bio-one, Germany and Corning Inc., NY, USA). Isolates from UTI are marked with dark blue as well as isolates from endocarditis (blue). Strains E. faecalis V583, OG1RF and OG1RFK12 (OG1RF including the PAI and plasmid pLG2 [45] were used as reference isolates as well as E. faecium 64/3 [54] as a negative control (all marked with black). Isolate UW7742 (= D32) is the completely sequenced ST40 reference isolate showing a lower biofilm forming capability. [file 12864_2015_1367_MOESM3_ESM.pptx]

## Slide 1
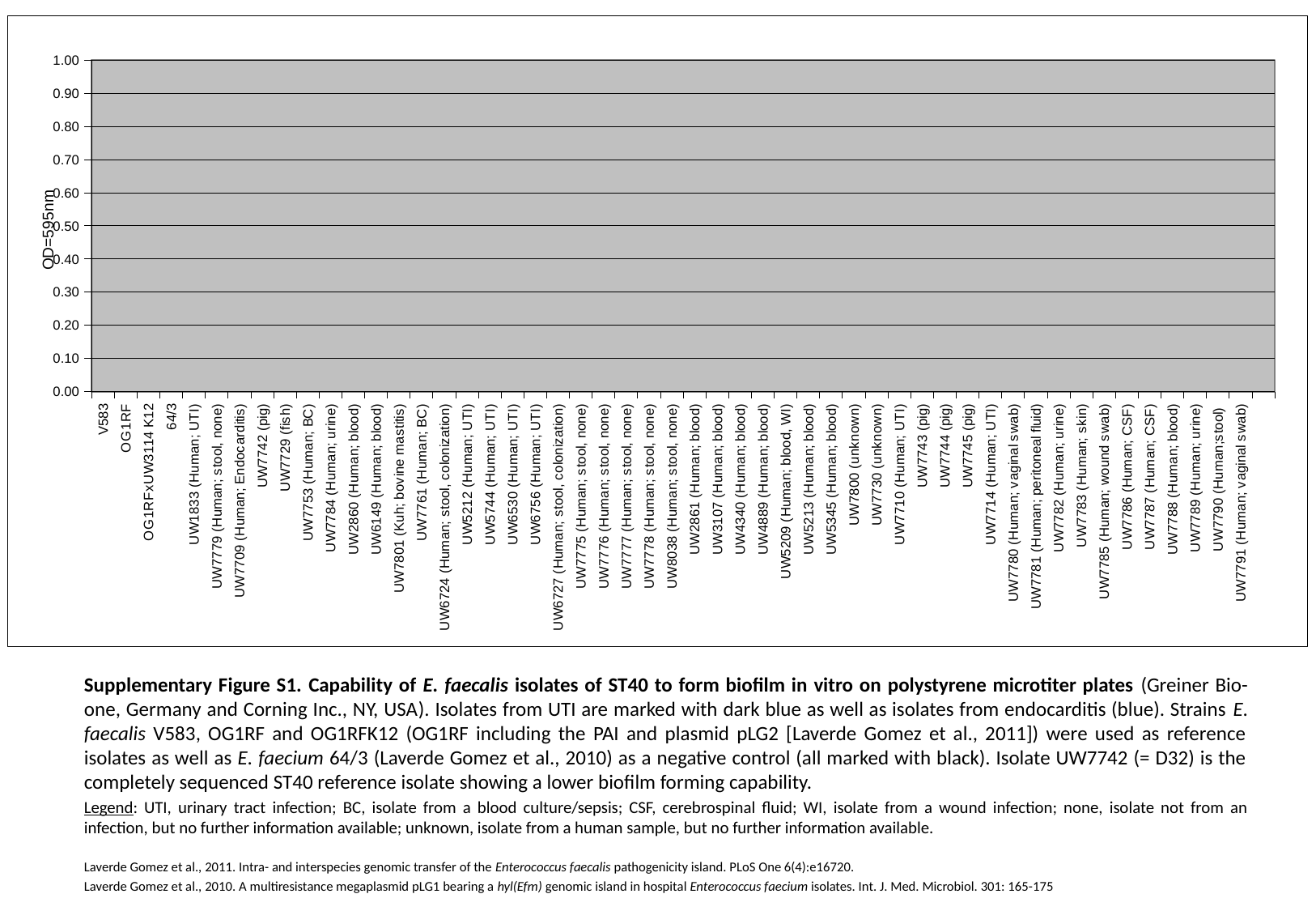

### Chart
| Category | |
|---|---|
| V583 | 0.32005611111111104 |
| OG1RF | 0.2265561111111111 |
| OG1RFxUW3114 K12 | 0.3076116666666666 |
| 64/3 | 0.017667222222222223 |
| UW1833 (Human; UTI) | 0.5554445555555555 |
| UW7779 (Human; stool, none) | 0.30700011111111114 |
| UW7709 (Human; Endocarditis) | 0.39911122222222223 |
| UW7742 (pig) | 0.16011122222222224 |
| UW7729 (fish) | 0.13655566666666663 |
| UW7753 (Human; BC) | 0.35755566666666666 |
| UW7784 (Human; urine) | 0.2550001111111111 |
| UW2860 (Human; blood) | 0.22722233333333333 |
| UW6149 (Human; blood) | 0.2446667777777778 |
| UW7801 (Kuh; bovine mastitis) | 0.19033344444444442 |
| UW7761 (Human; BC) | 0.25488900000000003 |
| UW6724 (Human; stool, colonization) | 0.20233344444444445 |
| UW5212 (Human; UTI) | 0.43833333333333335 |
| UW5744 (Human; UTI) | 0.573 |
| UW6530 (Human; UTI) | 0.47733333333333333 |
| UW6756 (Human; UTI) | 0.6040000000000001 |
| UW6727 (Human; stool, colonization) | 0.4336666666666667 |
| UW7775 (Human; stool, none) | 0.43333333333333335 |
| UW7776 (Human; stool, none) | 0.373 |
| UW7777 (Human; stool, none) | 0.17766666666666667 |
| UW7778 (Human; stool, none) | 0.535 |
| UW8038 (Human; stool, none) | 0.37533333333333335 |
| UW2861 (Human; blood) | 0.5533333333333335 |
| UW3107 (Human; blood) | 0.43333333333333335 |
| UW4340 (Human; blood) | 0.453 |
| UW4889 (Human; blood) | 0.33633 |
| UW5209 (Human; blood, WI) | 0.435 |
| UW5213 (Human; blood) | 0.55167 |
| UW5345 (Human; blood) | 0.46433 |
| UW7800 (unknown) | 0.646 |
| UW7730 (unknown) | 0.39867 |
| UW7710 (Human; UTI) | 0.47033 |
| UW7743 (pig) | 0.27567 |
| UW7744 (pig) | 0.163 |
| UW7745 (pig) | 0.16033 |
| UW7714 (Human; UTI) | 0.27533 |
| UW7780 (Human; vaginal swab) | 0.12767 |
| UW7781 (Human; peritoneal fluid) | 0.27733 |
| UW7782 (Human; urine) | 0.19833 |
| UW7783 (Human; skin) | 0.22466999999999998 |
| UW7785 (Human; wound swab) | 0.12567 |
| UW7786 (Human; CSF) | 0.43167 |
| UW7787 (Human; CSF) | 0.33067 |
| UW7788 (Human; blood) | 0.40333 |
| UW7789 (Human; urine) | 0.13833 |
| UW7790 (Human;stool) | 0.161 |
| UW7791 (Human; vaginal swab) | 0.12433 |Supplementary Figure S1. Capability of E. faecalis isolates of ST40 to form biofilm in vitro on polystyrene microtiter plates (Greiner Bio-one, Germany and Corning Inc., NY, USA). Isolates from UTI are marked with dark blue as well as isolates from endocarditis (blue). Strains E. faecalis V583, OG1RF and OG1RFK12 (OG1RF including the PAI and plasmid pLG2 [Laverde Gomez et al., 2011]) were used as reference isolates as well as E. faecium 64/3 (Laverde Gomez et al., 2010) as a negative control (all marked with black). Isolate UW7742 (= D32) is the completely sequenced ST40 reference isolate showing a lower biofilm forming capability.
Legend: UTI, urinary tract infection; BC, isolate from a blood culture/sepsis; CSF, cerebrospinal fluid; WI, isolate from a wound infection; none, isolate not from an infection, but no further information available; unknown, isolate from a human sample, but no further information available.
Laverde Gomez et al., 2011. Intra- and interspecies genomic transfer of the Enterococcus faecalis pathogenicity island. PLoS One 6(4):e16720.
Laverde Gomez et al., 2010. A multiresistance megaplasmid pLG1 bearing a hyl(Efm) genomic island in hospital Enterococcus faecium isolates. Int. J. Med. Microbiol. 301: 165-175
