# Supplementary material for: Comprehensive molecular, genomic and phenotypic analysis of a major clone of Enterococcus faecalis MLST ST40
Source: BMC Genomics. 2015 Mar 12;16(1):175. doi: 10.1186/s12864-015-1367-x (PMC4374294; doi:10.1186/s12864-015-1367-x)
Supplement: Additional file 4: Figure S2. — S1 nuclease analysis resolving plasmid content of 18 ST40 E. faecalis isolates. This representative gel of S1 nuclease PFGE showed the presence of none, one or two linearized plasmids. A red arrow points at a single plasmid band of the sequenced E. faecalis strain D32 (size of circa 75 kb). The upper bands (migrating above the 674 kb band of NCTC8325), visible in all lanes, correspond to undigested chromosomal DNA. NCTC8325 applies to SmaI-digested genomic DNA of S. aureus NCTC8325 used as a size marker in PFGE analysis. [file 12864_2015_1367_MOESM4_ESM.pptx]

## Slide 1
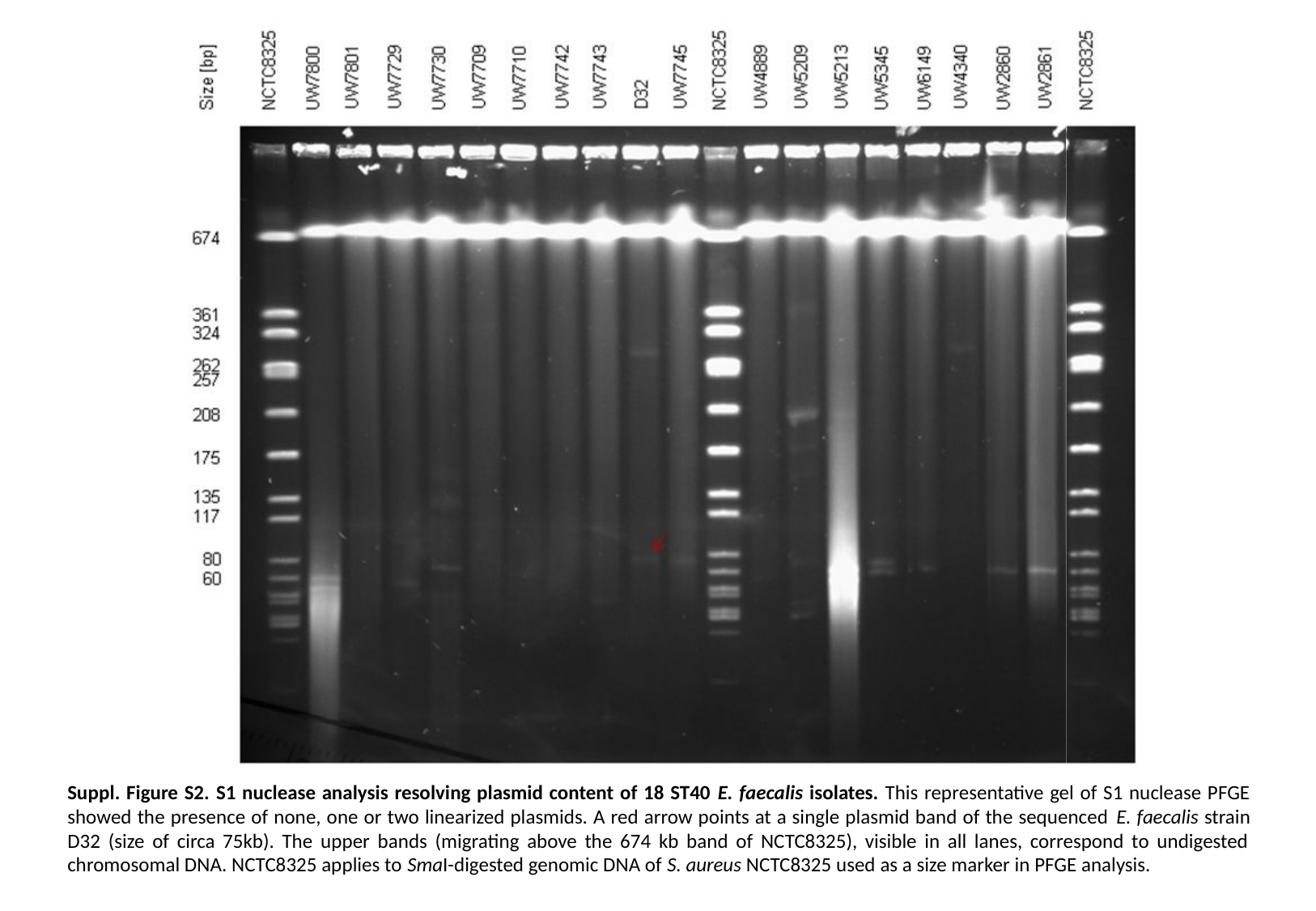

#
Suppl. Figure S2. S1 nuclease analysis resolving plasmid content of 18 ST40 E. faecalis isolates. This representative gel of S1 nuclease PFGE showed the presence of none, one or two linearized plasmids. A red arrow points at a single plasmid band of the sequenced E. faecalis strain D32 (size of circa 75kb). The upper bands (migrating above the 674 kb band of NCTC8325), visible in all lanes, correspond to undigested chromosomal DNA. NCTC8325 applies to SmaI-digested genomic DNA of S. aureus NCTC8325 used as a size marker in PFGE analysis.
