# Supplementary material for: Comprehensive molecular, genomic and phenotypic analysis of a major clone of Enterococcus faecalis MLST ST40
Source: BMC Genomics. 2015 Mar 12;16(1):175. doi: 10.1186/s12864-015-1367-x (PMC4374294; doi:10.1186/s12864-015-1367-x)
Supplement: Additional file 5: Figure S3. — Phylogenetic analysis of 15 E. faecalis ST40 genomes resulting from mapping illumina reads against the D32 reference genome. Solexa single reads were mapped onto the reference sequence of E. faecalis D32 by utilizing a mapping pipeline based on bwa (Steglich et al., previously unpublished [see Materials]). Altogether 10,4 % of ambiguous sites were detected compared to the reference genome and excluded from further analysis as well as SNPs resulting from recombination, thereby producing 1481 SNPs for phylogenetic calculations by the PhyML algorithm (seaview program) with a bootstrap of 1000. Metadata are given as follows: Strain no., year of isolation, origin, country: AC, animal colonizer; AI, animal infection; HC, human colonizer; HI, human infection; CU, Cuba; D, Germany; DK, Denmark; ES, Spain; IS, Island; PL, Poland; USA. [file 12864_2015_1367_MOESM5_ESM.pptx]

## Slide 1
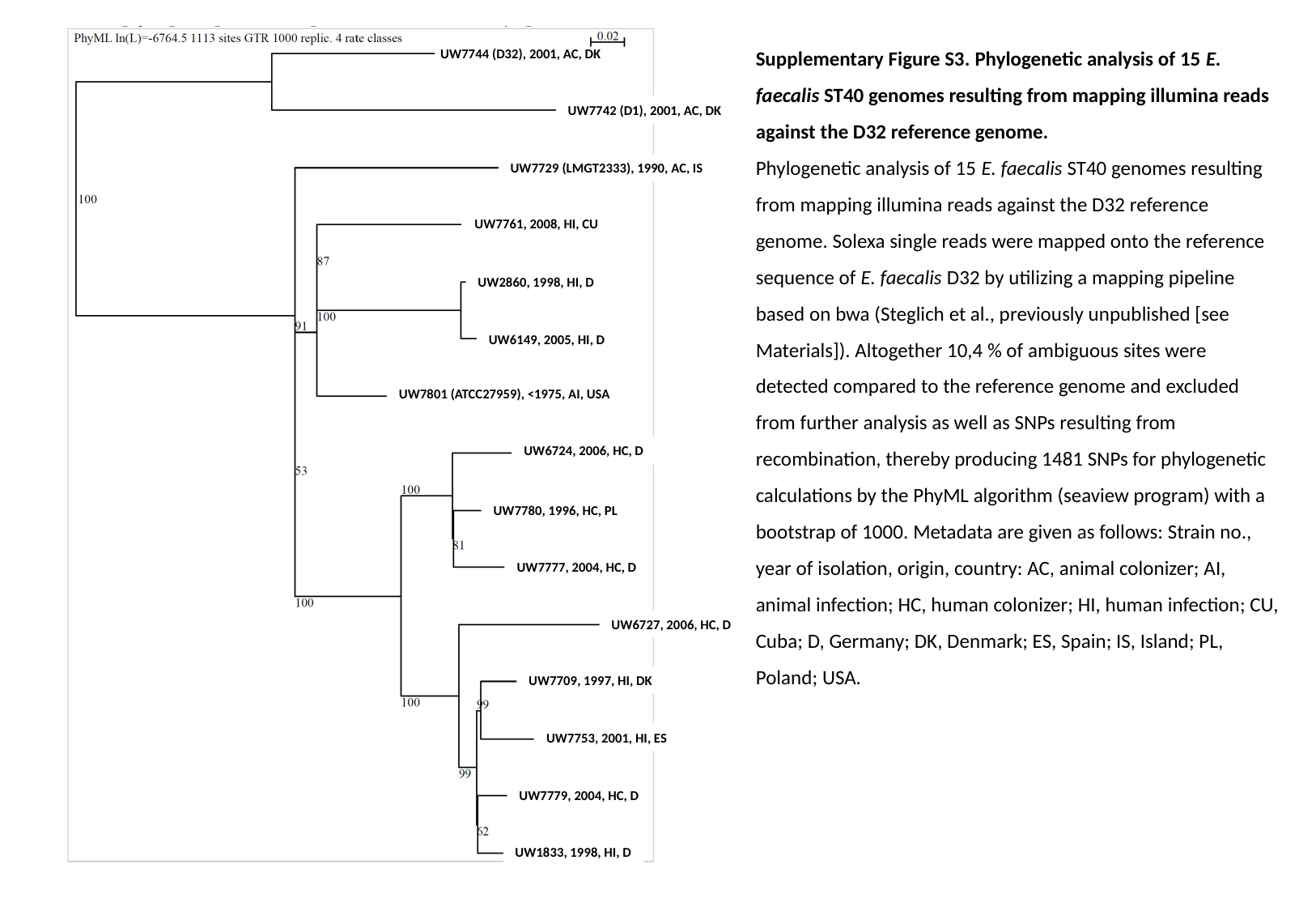

Supplementary Figure S3. Phylogenetic analysis of 15 E. faecalis ST40 genomes resulting from mapping illumina reads against the D32 reference genome.
Phylogenetic analysis of 15 E. faecalis ST40 genomes resulting from mapping illumina reads against the D32 reference genome. Solexa single reads were mapped onto the reference sequence of E. faecalis D32 by utilizing a mapping pipeline based on bwa (Steglich et al., previously unpublished [see Materials]). Altogether 10,4 % of ambiguous sites were detected compared to the reference genome and excluded from further analysis as well as SNPs resulting from recombination, thereby producing 1481 SNPs for phylogenetic calculations by the PhyML algorithm (seaview program) with a bootstrap of 1000. Metadata are given as follows: Strain no., year of isolation, origin, country: AC, animal colonizer; AI, animal infection; HC, human colonizer; HI, human infection; CU, Cuba; D, Germany; DK, Denmark; ES, Spain; IS, Island; PL, Poland; USA.
UW7744 (D32), 2001, AC, DK
UW7742 (D1), 2001, AC, DK
UW7729 (LMGT2333), 1990, AC, IS
UW7761, 2008, HI, CU
UW2860, 1998, HI, D
UW6149, 2005, HI, D
UW7801 (ATCC27959), <1975, AI, USA
UW6724, 2006, HC, D
UW6724, 2006, HC, D
UW7780, 1996, HC, PL
UW7777, 2004, HC, D
UW6727, 2006, HC, D
UW7709, 1997, HI, DK
UW7753, 2001, HI, ES
UW7779, 2004, HC, D
UW1833, 1998, HI, D
